# Gut microbiota and HMGB1/NLRP3/GSDMD inflammasome-dependent pyroptosis: mechanisms by physcion ameliorates alcoholic liver fibrosis

**DOI:** 10.3389/fphar.2025.1532590

**Published:** 2025-03-27

**Authors:** Ting Bai, Hao-Lin Guo, Fei Wang, Yan-Yu Kang, Hao-Tian Zhang, Lu Dong, Yong Yang

**Affiliations:** ^1^ Dalian Key Laboratory of Chronic Disease Research Center, Dalian University, Dalian, Liaoning, China; ^2^ Tangshan Seventh Hospital, Tangshan, Hebei, China

**Keywords:** alcoholic liver fibrosis, physcion, gut microbiota, HMGB1, NLRP3, cysteinyl aspartate specific proteinase-1 (Caspase-1), damage-associated molecular patterns (DAMPs), gasdermin D (GSDMD)

## Abstract

Alcoholic liver fibrosis (ALF) developed from long-term excessive alcohol consumption, which causes inflammatory reactions, lipid accumulation and cirrhosis. An imbalance in gut microbiota is a crucial driving factor for liver fibrosis through the gut-liver axis. This study aimed to explore the effect of physcion on ALF associated with HMGB1/NLRP3 pathways and gut microbiota. C57BL/6 mice were used to establish animal model of ALF, LX-2 cells were used to establish alcohol-activated cell model, the intestinal contents of the mice were collected and analyzed by 16S rRNA sequencing. Physcion effectively ameliorated ALF-induced inflammation, collagen deposition, lipid accumulation by SirT1, AMPK phosphorylation and SREBP1 expression. Moreover, pyroptosis-related proteins (Caspase-1, IL-1β, GSDMD) were significantly reduced after physcion treatment. Interestingly, the diversity of intestinal bacteria and the abundance in physcion treatment mice was significantly higher, while the abundance of harmful bacteria was significantly lower than that in ALF mice. Importantly, it was found that physcion inhibit HMGB1/NLRP3 pathways both *in vivo* and *in vitro*, and suppress accumulation of extracellular matrix by inhibiting Collagen-I and α-SMA to finally reverse hepatic stellate cells activation. Continuous administration of HMGB1 and NLRP3 inhibitors showed hepato-protection in alcohol-activated LX-2 model. siRNA-mediated knock-down in LX-2 cells of HMGB1 significantly impaired physcion-mediated protection. Regulation of the HMGB1/NLRP3 pathway recovered hepatic injury and further contributed to physcion’s beneficial effects. Taken together, the results reveal that physcion diminishes HMGB1/NLRP3 inflammasome/pyroptosis and that this diminishment is hepato-protective against ALF.

## Introduction

Alcoholic liver fibrosis (ALF) is developed from simple hepatic steatosis and steatohepatitis induced by alcohol consumption, which can further progress into cirrhosis and even hepatocellular carcinoma, posing a serious threat to human health (Mackowiak et al., 2024). As a result of the interaction of these factors, chronic inflammation of the liver leads to a series of pathophysiological processes, with the entire process of liver fibrosis involving different cells and mediators, abnormal growth of connective tissue in the liver, and persistent abnormal tissue growth leading to liver fibrosis (Liu N. et al., 2023). Thus, the mechanisms of liver fibrosis are complicated and involve different cells, signaling pathways, and cross-talk between individual cells.

High mobility group box 1 protein (HMGB1) is released extracellular as a stress signal and inflammatory mediator, when exogenous microorganisms invade or endogenous tissue damage occurs. HMGB1 is an evolutionarily conserved nuclear DNA-binding protein that is widely present in eukaryotic cells (Venereau et al., 2016). Once metastasized outside the cell, HMGB1 becomes a novel damage-associated molecular patterns (DAMPs) molecule and activates immune cells to produce tumor necrosis factor-α (TNF-α), interleukin-1β (IL-1β), and other pro-inflammatory factors, triggering an inflammatory response. HMGB1 has been found to play a key role in infection and aseptic inflammation, inducing pyroptosis by binding to RAGE or Toll receptor 4 (TLR4) (Paudel et al., 2020). NOD-like receptor protein 3 (NLRP3) is the most well-known member of the NLR family and has been extensively studied in liver disease (Torres et al., 2022). The assembly of NLRP3 inflammasomes begins with cytosolic pattern recognition receptors (PRRs), which recognize pathogen-associated molecular patterns (PAMPs) and DAMPs, which then signal recognition to apoptosis-associated spot proteins (ASCs) to form intracellular macromolecular protein complexes (de Carvalho Ribeiro and Szabo, 2022). This in turn promotes a range of inflammatory responses. Recent research has shown the crucial role of pyroptosis in alcohol-associated liver disease (ALD) mediated by inflammasomes (Gan et al., 2022). It seems that the NLRP3 inflammasome in hepatocytes may play a pivotal role in developing liver fibrosis.

Pyroptosis is a distinct type of programmed cell death characterized by the formation of cell membrane pores, the release of intracellular contents, nuclear condensation, and cell lysis (Brahadeeswaran et al., 2023). Pyroptosis is seen not only in monocytes, macrophages, but also in hepatocytes, dendritic cells and others (Gaul et al., 2021). As more frequent reports of pyroptosis have been reported, unlike programmed cell necrosis, pyroptosis initiates the NLRP3 inflammasome, which converts the pro-cysteinyl aspartate specific proteinase-1 (Caspase-1) precursor to activated Caspase-1 with inflammatory infiltration. At the same time, activated caspases cleave the gasdermin protein (GSDMD), and the pores that form on the cell membrane led to the loss of membrane integrity, the release of cell contents, and ultimately the occurrence of pyroptosis (Rao et al., 2022).

Various liver diseases are characterized by an impaired gut barrier and a disturbed gut-liver axis. The anatomical structure of the liver determines its inseparable role with the gut, and the nutrients and microbiota in the intestine contribute to maintain the metabolic activity of liver health (Tilg et al., 2022). In this instance, the liver is overwhelmed by antigens, metabolites, and possibly gut microbes, which potentially aggravate pre-existing liver disease through liver inflammation (Trebicka et al., 2021). In ALF, the interaction between the microbiome and the host liver is of particular concern, and there has been evidence that alcohol alters the composition of the gut microbiota and impairs gut integrity and barrier function (Bajaj, 2019). Restoring the intestinal microenvironment may be a potential and effective way to ameliorate liver injury (Zhu et al., 2022).

**FIGURE 1:**
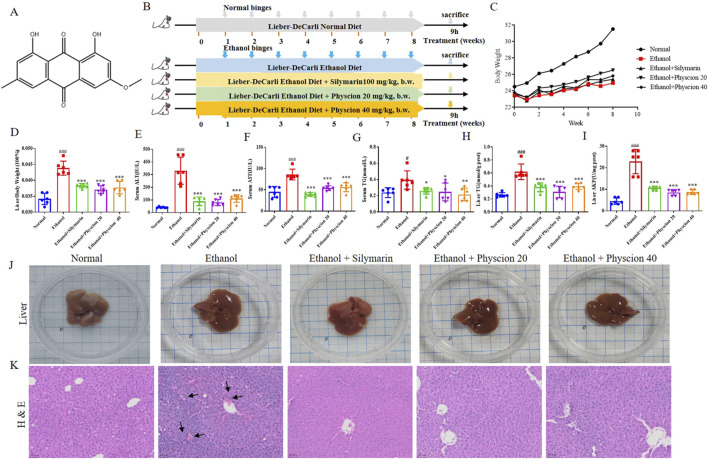
Effect of physcion on hepatic injury after chronic-binge ethanol consumption in mice. **(A)** Physcion structure; **(B)** Flow chart of ALF model; **(C)** Body weight in mice; **(D)** Liver-to-body weight ratio; **(E)** Serum ALT activities; **(F)** Serum AST activities; **(G)** Serum TG levels; **(H)** Liver TG levels; **(I)** Liver AKP activities; **(J)** Mouse liver appearance; **(K)** H&E staining (The black arrows represent bruises, scale bars 50 µm). Data are presented as the mean ± SD (*n* = 6). Significant differences between the indicated groups: ^#^ *P* < 0.05, ^###^ *P* < 0.001 vs. Normal group; *P < 0.05, **P < 0.01, ***P < 0.001 vs. Ethanol group.

Physcion is a natural anthraquinone derivative, which mainly reported in Rhubarb (Rheum sp.) (Liu X. et al., 2023). The structural is shown in Figure 1A. As the major active ingredients, anthraquinone derivatives have been documented as anti-cancer, anti-inflammatory, antifungal and antiviral effects (Trybus et al., 2021; Liu et al., 2024). Recent reports indicate that physcion protects the liver from ethanol-induced injury by resetting the circadian clock (Yao et al., 2020). However, the literature lacks data of physcion on gut microbiota and ALF, especially with regard to the HMGB1/NLRP3 pathway and the related pyroptosis process. We aimed to explore whether the protective effect of physcion is associated with the HMGB1/NLRP3 pathways and the regulation of gut microbiota, which would provide new ideas for drug treatment of ALF.

## Materials and methods

### Chemicals and reagents

Physcion (HY-N0108, >99%), Glycyrrhizic acid (GA, HY-N0184), MCC950 (HY-12815A) were purchased from Med Chem Express (Shanghai, China). Silymarin (S0292) was purchased from Sigma-Aldrich. Lieber-DeCarli liquid feed (TP4030)was purchased from Trophic Animal Feed High-Tech Co., Ltd., (Jiangsu, China). Primary antibodies against Collagen-Ⅰ (#ab88147), NLRP3 (#ab263899), GSDMD (#ab219800), SREBP1 (#ab28481), anti-Escherichia coli (*Escherichia coli*, #ab20640) were purchased from Abcam (Cambridge, MA, United States). GAPDH (#2118), α-Smooth Muscle Actin (α-SMA, #19245), HMGB1 (#6893), IL-1β (#12242), IL-18 (#54943), SirT1 (#2028), AMPKα (#5831), Phospho-AMPKα, (p-AMPKα, #2535), Acetyl-CoA Carboxylase (ACC, #3676), Phospho-Acetyl-CoA Carboxylase (p-ACC, #11818), Anti-Mouse IgG (#7076), Anti-Rabbit IgG (#7074) were purchased from Cell Signaling Technology (Beverly, MA, United States). F4/80 (#sc-377009), Caspase-1 (#sc-392736), control siRNA (#sc-37007), HMGB1-siRNA (#sc-37982) were purchased from Santa Cruz Biotechnology, Inc., (CA, United States). PrimeScript™ II 1st Strand cDNA Synthesis Kit (6210) was purchased from TaKaRa Biotech Inc., (Beijing, China).

### Animal model and experimental design

Male C57BL/6N mice aged 6–8 weeks (20 ± 2 g), SPF, were collected from Liaoning Changsheng Biotechnology Co., Ltd., (Liaoning, China) [SPF, SCXK (L) 2004-0017]. Mice were housed two to three per cage in the individually ventilated cage System at the Dalian University Experimental Animal Center in laboratory environments (22°C ± 2°C, 45% ± 5% relative humidity, 12 h light/dark cycle). All animal breeding and experimental procedures were performed in accordance with the Guide for the Care and Use of Laboratory Animals and were approved by the Ethics Committee of Zhongshan Hospital Affiliated to Dalian University (Protocol number DWLL2019060) and the ARRIVE guidelines.

Long-term chronic plus multiple binge ethanol feeding model induction was established as previously reported, with modifications to assess the effect of physcion on ALF development (Ramirez et al., 2017). The process of establishing the animal model is detailed in Figure 1B. Briefly, adaptive feeding was undertaken for 1 week, then animals were randomly divided into five groups, with six mice per group. The mice were specifically divided into normal (pair feeding, Normal) group, model (alcohol feeding, Ethanol) group, positive control (silymarin 100 mg/kg, b.w.) group, low-dose physcion treatment (Ethanol + Physcion 20) group and high-dose physcion treatment (Ethanol + Physcion 40) group. Mice in the normal group were fed control Lieber-DeCarli liquid diet, mice in the ethanol group and ethanol + physcion groups were fed 5% (v/v) ethanol Lieber-DeCarli liquid diet, with the ethanol concentration gradually increasing from 1% (v/v) to 5% (v/v) within the first 7 days and then maintained at 5% (v/v) ethanol for the next 8 weeks; during administration, ethanol- and pair-fed mice are gavaged once a week throughout the 8-week chronic feeding period with a single dose of ethanol (5 g/kg, b.w.) or isocaloric maltodextrin (9 g/kg, b.w.), respectively. During administration (week 1 through week 8), mice in the physcion-treated groups were gavaged with 20/40 mg/kg b.w. physcion solution, and mice in the normal group and ethanol group were gavaged with equivalent volume of normal saline solution. Mice are euthanized 9 h after the last gavage. The serum was collected, liver tissue was separated, the left anterior lobe of the liver was fixed with paraformaldehyde for subsequent pathological detection, and the remaining liver tissue was frozen at −80°C for RT-PCR and protein extraction experiments. The intestinal contents of the sacrificed mice were collected and analyzed by the 16S rRNA sequencing method to assess the composition of intestinal bacteria.

### Biochemical analysis and assay of mouse liver index

Blood samples were collected after eyeball removal, and the serum was prepared by centrifugation (1,000×g, 10 min). Serum alanine aminotransferase (ALT), aspartate aminotransferase (AST) and triglyceride (TG) kits were measured using the commercial kit (Nanjing Jiancheng Bioengineering Institute, Jiangsu, China). Liver homogenates were prepared from liver tissues with ice-cold saline (1/9, w/w), liver alkaline phosphatase (AKP) and TG concentrations were determined using the respective detection kits according to the manufacturer’s instructions. The mouse liver index was calculated according to the following Eq.: Liver index = weight of the liver/body weight.

### Liver histomorphology and immunohistochemistry

Fix mouse liver tissue in 4% paraformaldehyde and then embed it in paraffin. Prepare 5 µm thick liver tissue sections and stained with hematoxylin-eosin (H&E) and Masson. Immunohistochemistry (IHC) for the liver tissue with the primary antibody as previously described (Alexander et al., 2018). The inflammation and fibrosis of the liver were observed under the light microscope. Frozen sections (5 μm) were thawed to room temperature for Oil Red O staining and microscopic observation of liver lipid accumulation. Quantitative statistics of staining were obtained using ImageJ software (Media Cybernetics, MD, United States).

### Immunofluorescence

Paraffin-fixed sections (5 μm) were pre-pared on slides, deparaffinized in xylene and endogenous peroxidase activities were blocked by incubation in a 3% H_2_O_2_ solution at room temperature for 10 min. Antigen retrieval was performed for 30 min. Incubation with primary antibodies were performed at 4°C overnight. Secondary antibody incubation was performed for 1 h at room temperature. The DAPI staining was conducted according to the protocol provided by the manufacturer.

The cells were permeabilized with 0.1% Triton X-100 in PBS for 20 min following 15 min of fixation in 4% paraformaldehyde. The membrane was then blocked with 1% BSA for 1 h at room temperature. After that, the cells were incubated with primary antibodies at 4°C overnight. The cells were incubated with the appropriate secondary antibodies for 1 h at room temperature and then counterstained with DAPI. Images were captured with an Olympus microscopy imaging system at 400× magnification.

### 16S rRNA gene sequencing and analysis

The feces collected from ALF mice and frozen at −80°C for analyzing by 16S rRNA sequencing to evaluate the composition of intestinal bacteria. A detailed version of the protocol can be found in previous publications (Jia et al., 2024). Microbial DNA was extracted from intestinal contents using Stool DNA extraction kits (Omega, United States) according to the manufacturer’s protocols. The 16S rRNA V3-V4 regions were amplified using specific primers and sequencing was conducted on Illumina MiSeq platform (LC-Bio Technology, China). The feature sequences were used to calculate alpha diversity, beta diversity, the Shannon index and Chao index with QIIME2 and further annotated with the SILVA database. LEfSe (p < 0.05) was performed using the online tool at https://www.omicstudio.cn. Heatmaps and other diagrams were implemented using R package (v3.5.2.).

### Cell culture and transfection

The human hepatic stellate cell line, LX-2 and the mouse monocytic macrophage cells, RAW264.7 were purchased from EK-Bioscience (Shanghai, China). Cells were kept with 5% CO_2_ incubator in DMEM medium supplemented with 10% FBS.

To induce *in vitro* ALF-like injury of LX-2 cells, we co-treated cells with 50 mmol/L ethanol and 5 ng/mL transforming growth factor-β (TGF-β, Med Chem Express, #HY-P73616) for 6 h. To investigate the effects of physcion on LX-2 cells, different doses of physcion were added along with concurrent TGF-β and ethanol for a 6-h incubation.

LX-2 cells were incubated in Opti-DMEM and transfected with Control or HMGB1-specific siRNA (100 nM) using HiPerFect Transfection Reagent (QIAGEN, 301705), according to the manufacturer’s protocol. Following the transfection, ethanol and physcion were added and co-incubated in the same way as the above procedures. After the treatments, cells were harvested for subsequent experiments.

### Protein extraction and Western blot

After treatments, cells or liver tissues were lysed in RIPA buffer (Solarbio, Beijing, China) and separated on SDS-PAGE, then electroblotted onto PVDF membrane and incubated overnight with the indicated antibodies at 4°C. After incubation with the secondary antibody, the protein bands are collected using chemiluminescence imaging system. Process all samples in the same group at the same time and the protein expression was homogenized to GAPDH.

### RNA extraction and reverse transcription polymerase chain reaction (RT-PCR)

Total RNA was extracted from liver tissue samples and LX-2 cells using TRNzol Universal Reagent (TIANGEN Biotech, Beijing, China). cDNA was synthesized from 1 μg of total RNA using the PrimeScript™ II 1st Strand cDNA Synthesis Kit following the manufacturer’s instructions. The amplification conditions were used: 95°C for 30 s, 95°C for 5 s, and 35 cycles at 60°C for 5 s. The levels of mRNA were normalized in relation to endogenous GAPDH. The prime sequence (Sangon Biotech, Shanghai, China) of related genes is shown in Supplementary Table S1.

### Statistical analysis

All data were expressed as mean ± standard (SD) deviation using GraphPad Prism 9.0 software. At least five independent replications were performed. Data were analyzed by one-way analysis of variance (ANOVA), followed by Tukey’s multiple comparisons test. *P* values of less than 0.05 denoted statistically significant.

## Results

### Physcion ameliorated hepatic injury after chronic-binge ethanol consumption

In order to prove the protective effect of physcion on hepatic injury of chronic ethanol consumption, we first recorded the changes in the body weight of the mice during the experiment. The weight of the mice in the alcohol diet group decreased significantly, yet the body weight of mice was significantly improved after physcion and silymarin treatment (Figure 1C). The chronic-binge ethanol significantly increased the liver weight, liver-to-body ratio, serum levels of ALT, AST and liver AKP which indicating a more severe status of hepatic injury, and the markedly elevated levels of TG suggesting a state of dysregulated lipid metabolism (Figures 1D–I). Co-treatment with physcion and silymarin effectively ameliorated those abnormalities without changing the basal conditions. This was further validated by liver status and histology (Figures 1J, K). In the normal group, the surface of the liver was reddish-brown whereas the surface of the liver in the ethanol group was rough and dull. H&E staining showed that the ethanol mice had obvious edema in the liver, destruction of liver lobules, mononuclear cell infiltration, obvious fat vacuoles in hepatocytes, and disordered lobular structure. In comparison, in the physcion groups, the hepatic lobular structure was basically restored to normal, and the hepatic cord was gradually clear. These results suggest that physcion can alleviate chronic liver injury, steatosis, and liver inflammation induced by chronic ethanol consumption.

### Physcion ameliorated the development of alcohol-induced liver fibrosis

**FIGURE 2:**
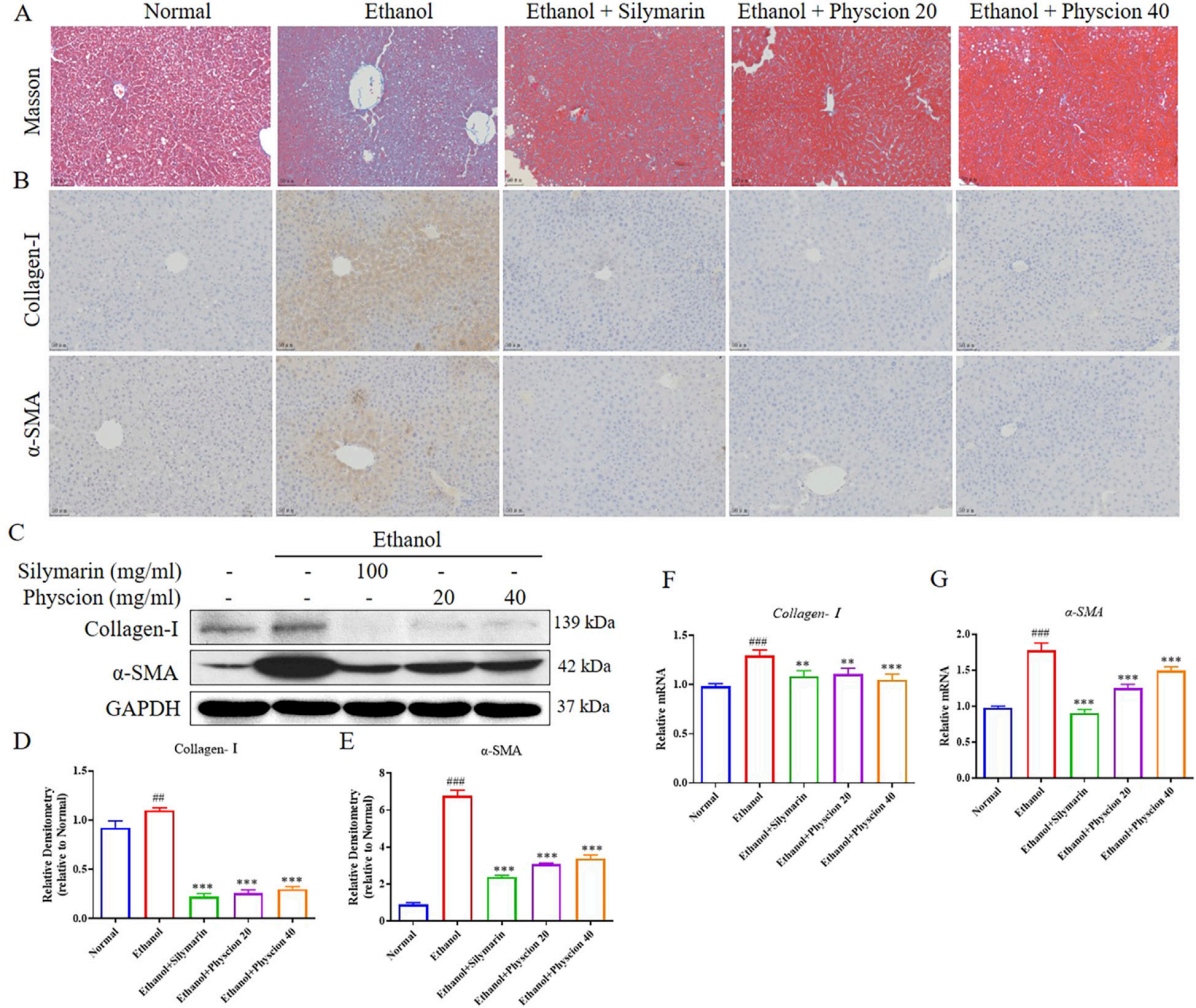
Effect of physcion on the development of alcohol-induced liver fibrosis. **(A)** Masson staining; **(B)** IHC was used to measure the levels of Collagen-I and α-SMA; **(C–E)** The protein expression levels and relative protein expression statistics of Collagen-I and α-SMA; **(F, G)** The mRNA level of Collagen-I and α-SMA. Scale bars 50 µm. Significant differences between the indicated groups: ^##^ *P* < 0.01, ^###^ *P* < 0.001 vs. Normal group; **P < 0.01, ***P < 0.001 vs. Ethanol group.

Liver fibrosis is usually accompanied by changes in the expression of fibrosis-related factors. To explore the degree of liver injury in drinking mice, we further tested the following indicators. Masson staining (Figure 2A) showed that, the ethanol group had massive proliferation of fibrous tissue surrounding the central vein and confluent area of the liver, along with a notable increase in collagen deposition, appeared bridging fibers, formed obvious vascular fiber intervals, and numerous perisinusoidal fiber bundles with a wire mesh-like structure were observable. After physcion treatment, the collagen fiber hyperplasia in the liver of mice were significantly reduced and the liver morphology returned to normal. IHC (Figure 2B) showed that the biomarkers of activated HSCs, including Collagen-Ⅰ and α-SMA, the brown-yellow positive area in the liver tissues of the ethanol group were significantly higher than those of the normal group. After treatment with physcion and silymarin, the liver immunohistochemical staining positive area of mice was significantly relieved. At the protein and mRNA levels (Figures 2C–G), the expression of liver fibrosis-related factors (Collagen-Ⅰ and α-SMA) increased in the ethanol group, and physcion and silymarin could significantly alleviate the development of fibrosis.

### Physcion alleviated alcohol-induced gut microbiota disorders in mice

**FIGURE 3:**
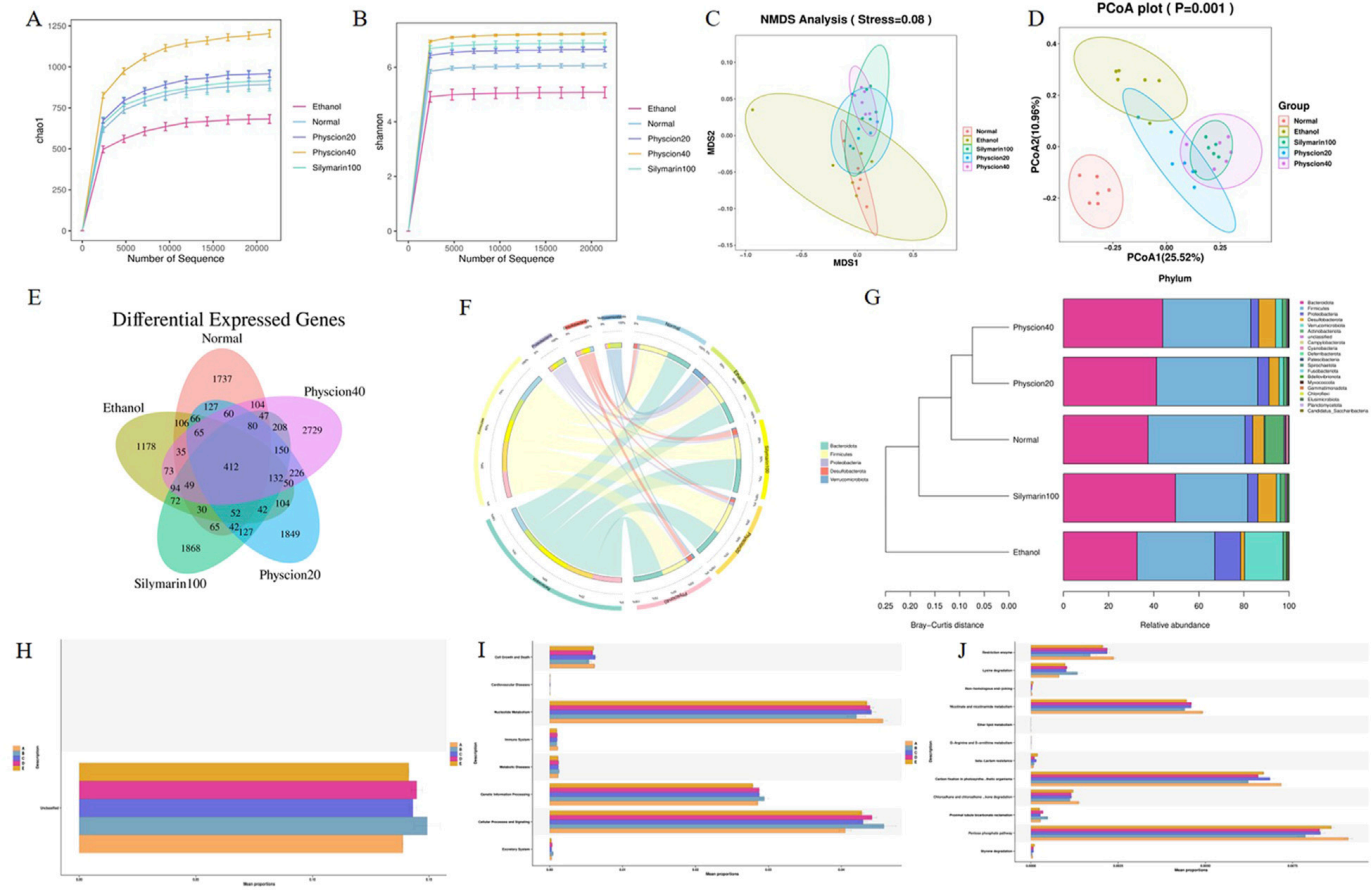
Effect of physcion on gut microbiota disorders in mice at portal level. **(A)** Chao1 analysis; **(B)** Shannon analysis; **(C)** NMDS analysis; **(D)** PCoA analysis; **(E)** Venn analysis; **(F)** Circos map of the intestinal microbiome of mice in each group; **(G)** Species diversity analysis; **(H–J)** Function analysis. Abbreviation: A = Normal group, B = Ethanol group, C = Ethanol + Silymarin group, D = Ethanol + Pyhscion 20 group, E = Ethanol + Pyhscion 40 group.

Studies have found that gut microbes can participate in the metabolism of human substances, are the components of biological barriers, and are closely related to immune regulation and host defense functions (Hsu and Schnabl, 2023). Therefore, we examined the intestinal contents of each group of mice during ALF. The dilution curves of Chao1 and Shannon diversity index of mouse intestinal fecal samples showed a gentle trend with the increase of sample size (Figures 3A, B), indicating that the sequencing data of our samples were reasonable, the sample size was large enough, the sample uniformity was good enough to contain most bacterial species. NMDS and PCoA (Figures 3C, D) results showed significant differences in the composition of intestinal flora between the normal group and the ethanol group. After treatment with physcion, the intestinal flora disorder was improved, and the effect was similar to that of silymarin group. According to the Venn diagram (Figure 3E), the diversity of intestinal flora in mice was significantly reduced due to the effect of alcohol, and the diversity of intestinal flora in mice was restored after treatment with physcion and silymarin.

Through Circos diagram analysis of alcohol-induced intestinal dominant flora of mice, there were different bacterial taxa in the intestinal flora of mice in each group (Figure 3F). In terms of phylum level, the intestinal flora of normal mice is mainly composed of *Firmicutes* and *Bacteroidota*, which belong to the dominant phylum, with firmicutes slightly more than *Bacteroides* and *Desulfobacterota*. However, *Proteobacteria*, *Verrucomicrobiota* and *Actinobacteriota* occupy a small proportion in the intestines of normal mice. Compared with the ethanol group, the proportion of *Bacteroides* and *Firmicutes* decreased significantly, while the proportion of some harmful bacteria such as *Verrucomicrobiota* and *Proteobacteria* increased significantly. After the physcion and silymarin treatment, the intestinal flora disorder of mice was reversed. In addition, cluster analysis showed that the intestinal flora of mice treated with physcion was more similar to normal mice (Figure 3G).

PICRUSt2 was used to predict the function of the bacterial community in the intestinal tract of mice, and the functional abundance prediction table and the metabolic pathway abundance table were obtained by referring to the KEGG database. The results showed that there was no significant difference in gene abundance in the primary functional layer (Figure 3H), while there were differences in the abundance of functional genes in the secondary functional layer, especially in metabolism and cellular processes and signaling pathways (Figure 3I). It was further explored that there were significant differences in the tertiary functional layer, especially in the pentose phosphate pathway, niacin and nicotine metabolism, and lysine metabolism pathway (Figure 3J). The metabolism of nicotinate and nicotinamide was weakened in the ethanol model group, while the degradation ability of lysine was increased, which aggravated the accumulation of liver fat and promoted the progression of ALF. After treatment with physcion and silymarin, the above changes were significantly alleviated.

**FIGURE 4:**
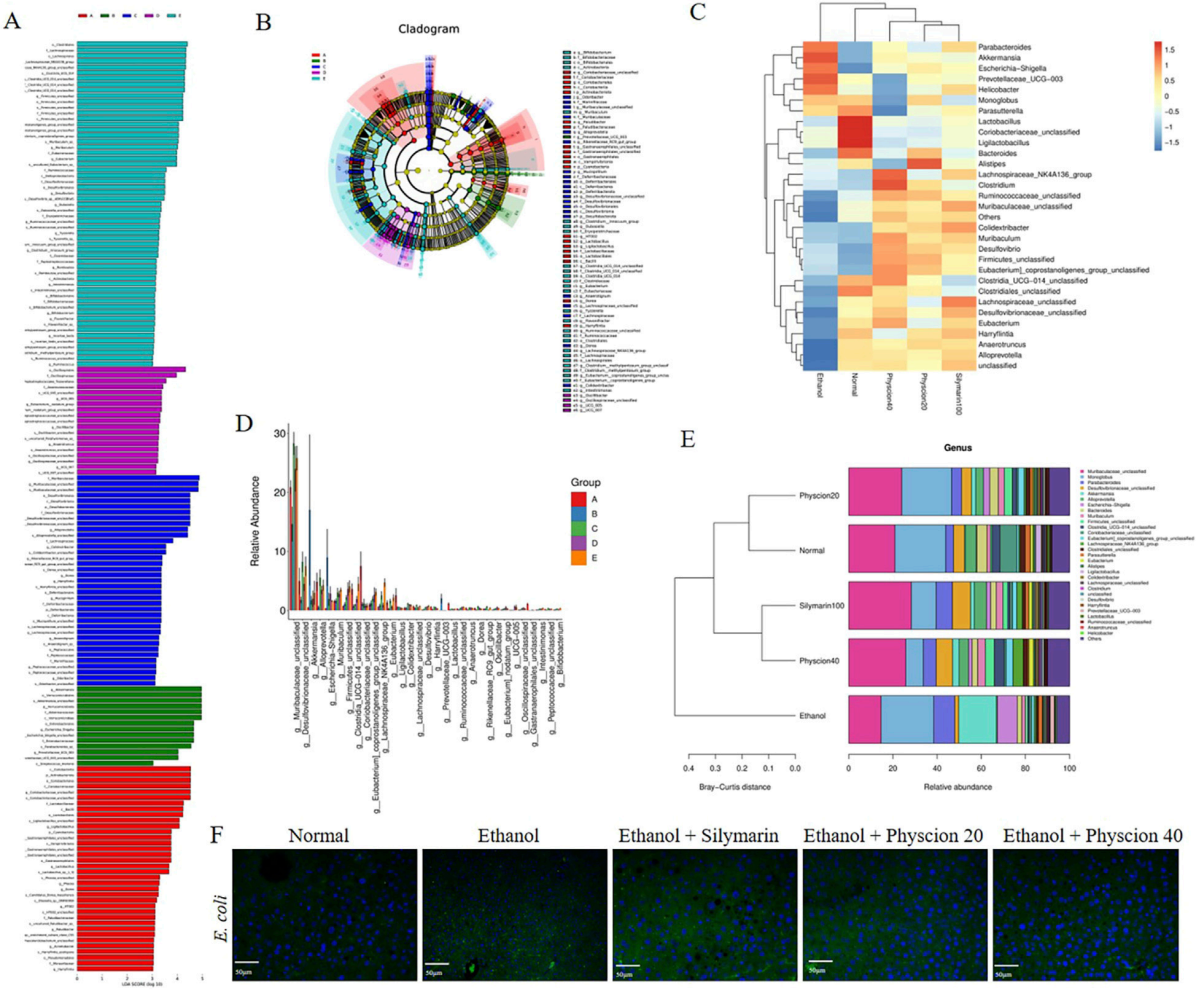
Effect of physcion on gut microbiota disorders in mice at the generic level. **(A, B)** LEfSe analysis of mice in each group; **(C)** Heat map analysis of mice in each group; **(D)** Difference analysis of mice in each group; **(E)** Species diversity analysis of mice in each group; **(F)** Liver immunofluorescence staining of *Escherichia coli* in each group (scale bars 50 µm). Abbreviation: A = Normal group, B = Ethanol group, C = Ethanol + Silymarin group, D = Ethanol + Pyhscion 20 group, E = Ethanol + Pyhscion 40 group.

At the genes level, the LEfSe diagram analysis of the intestinal flora of mice (Figures 4A, B) showed the same results as before. *Desulfovibrionaceae*, *Muribaculaceae* and *Alloprevotella* are significantly reduced in the intestinal bacteria compared with the normal group. *Desulfovibrionaceae*, *Akkermansia* and *Escherichia_Shigella* have increased significantly (Figures 4C, D). After physcion treatment, the changes in the intestinal flora of mice were reversed, and cluster analysis showed that the distribution of intestinal bacteria in the physcion groups were more similar to that in the normal group (Figure 4E). In addition to affecting the diversity and richness of intestinal flora in mice, alcohol metabolites can also destroy intestinal barrier function, resulting in bacterial displacement. We conducted immunofluorescence detection of *E. coli* in liver, and the fluorescence signal intensity of *E. coli* in the ethanol group was significantly higher than that in the normal group, but the increase trend was inhibited in the physcion groups (Figure 4F). These results indicate that physcion alleviate alcohol-induced intestinal flora disturbance in mice.

### Physcion relieved alcohol-induced liver steatosis

**FIGURE 5:**
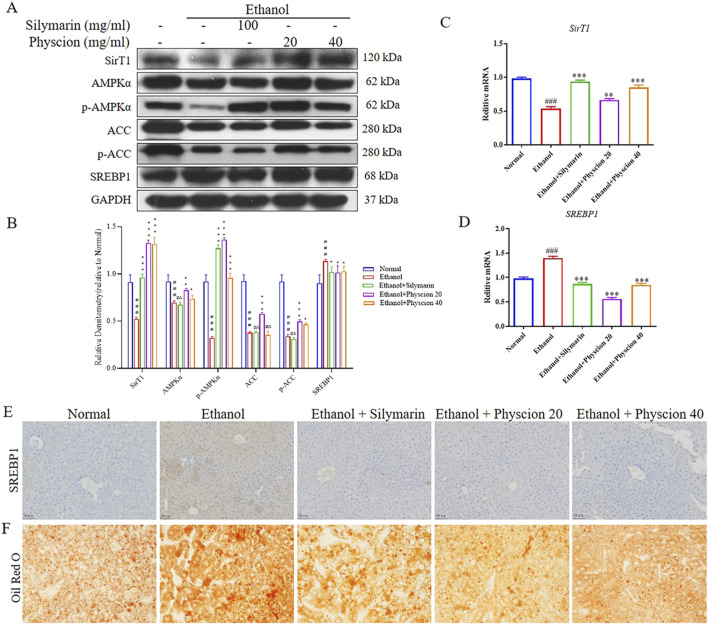
Effect of physcion on alcohol-induced liver steatosis. **(A, B)** The protein expression levels and relative protein expression statistics of SirT1, AMPKα, p-AMPKα, ACC, p-ACC and SREBP1; **(C, D)** The mRNA levels of SirT1 and SREBP1; **(E)** IHC was used to measure the levels of SREBP1; **(F)** Oil Red O staining of the liver. Scale bars 50 µm. Significant differences between the indicated groups: ^###^ *P* < 0.001 vs. Normal group; *P < 0.05, **P < 0.01, ***P < 0.001 vs. Ethanol group.

After alcohol consumption, catabolism in the body can inhibit or activate related transcription factors in various ways (Quan et al., 2013). To further prove the role of physcion in ethanol induced hepatocyte protection, we characterized the expressional changes of lipid metabolism-related markers SREBP1, AMPK, ACC and SirT1 after ethanol and physcion treatments. Alcohol exposure significantly reduced SirT1, phosphorylation of AMPKα and ACC, as expected, co-treatment with physcion restored the reductions. Importantly, mice fed with ethanol exhibited upregulated SREBP1 protein expression and mRNA levels, physcion treatment significantly ameliorated such transitions (Figures 5A–D). At the same time, IHC staining was used to determine the effects of physcion and silymarin on the expression of SREBP1 in the liver, and the results showed that SREBP1 were positive for yellow or brownish yellow. The expression of SREBP1 in ethanol group was significantly taller, and after physcion treatment, SREBP1 was significantly reduced and had the same effect as the silymarin group (Figure 5E). The Oil Red O staining will burn the lipids in the cells to a red color. The ethanol group had severe fat accumulation in the liver tissue, and silymarin and physcion were able to significantly reverse this situation (Figure 5F). These results suggest that physcion alleviate alcohol-induced liver steatosis in mice by reducing lipogenesis.

### Physcion suppressed liver inflammation via HMGB1 related signaling pathways

**FIGURE 6:**
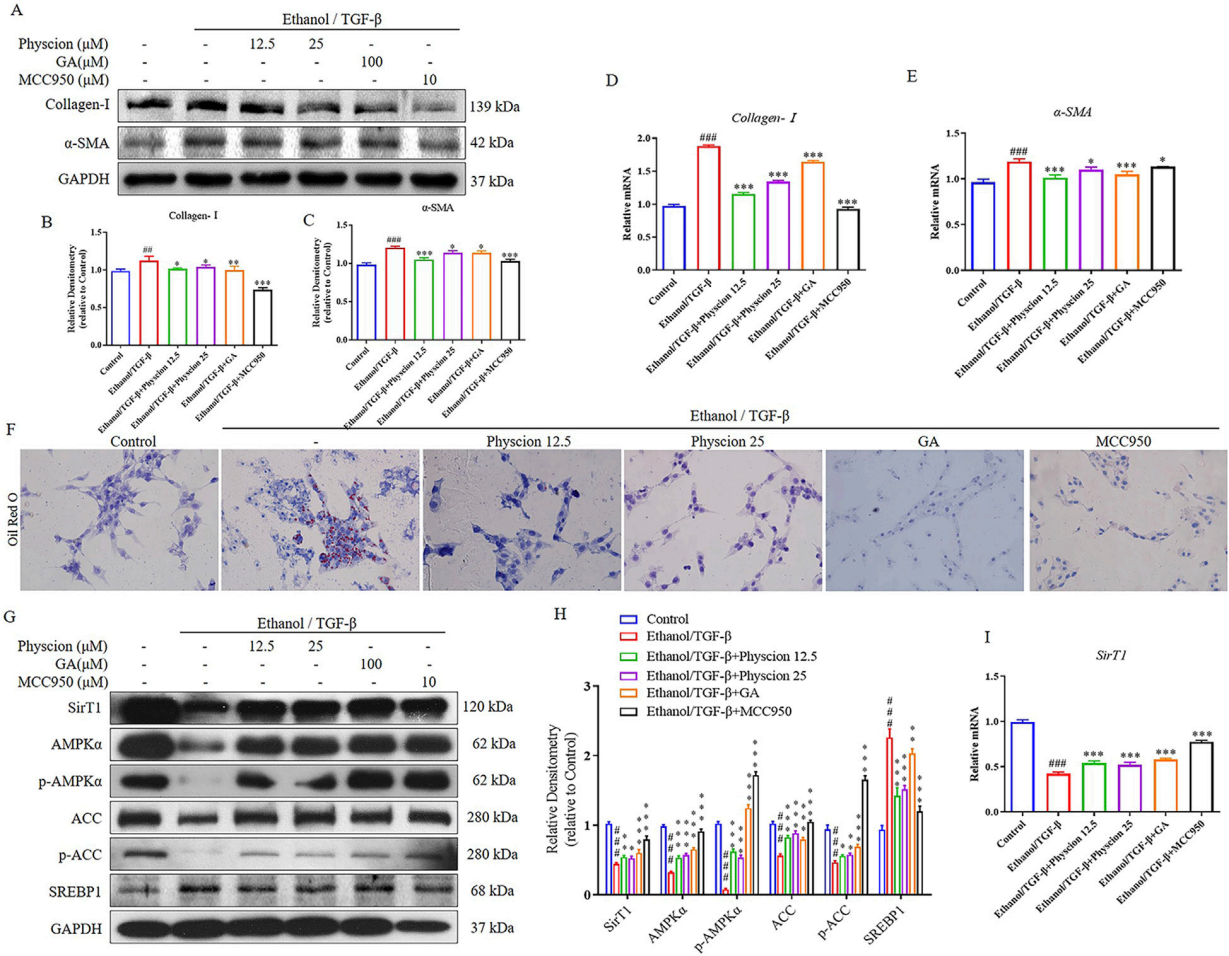
Effect of physcion on liver inflammation via HMGB1 related signaling pathways. **(A, B)** The protein expression levels and relative protein expression statistics of HMGB1, NLRP3, cleaved-Caspase-1, mature IL-1β, IL-18 and GSDMD; **(C–H)** The mRNA levels of HMGB1, NLRP3, Caspase-1, IL-1β, IL-18 and GSDMD; **(I)** Liver immunofluorescence staining of HMGB1, NLRP3 and GSDMD in liver (scale bars 50 µm). Significant differences between the indicated groups: ^#^ *P* < 0.05, ^###^ *P* < 0.001 vs. Normal group; **P < 0.01, ***P < 0.001 vs. Ethanol group.

It is worth noting that inflammasome activation and pyroptosis play a central role in the inflammatory cascade, which induces HSCs activation and eventually leads to liver fibrosis (Gan et al., 2022). Hereby, we detected the expression of liver-related inflammatory factors in ALF mice. Western blot found the increased expression of HMGB1, NLRP3, Caspase-1, IL-1β, IL-18 and GSDMD in liver when compared with those in the normal group. Physcion and silymarin treatment obviously suppressed the expression of inflammatory factors in mice (Figures 6A, B). In addition, at the mRNA level, the same validation was obtained (Figures 6C–H). In immunofluorescence analysis (Figure 6I), the effect of physcion on the expressions of HMGB1, NLRP3 and GSDMD in the liver were confirmed the above results again. Physcion groups significantly reduced the protein expressions, and showing the same therapeutic effect as silymarin group. Accordingly, HMGB1 signaling pathway might act on chronic-binge ethanol consumption induced liver injury and liver inflammation attenuated by physcion in ALF mice through inhibiting HMGB1/NLRP3/GSDMD pathway.

### Physcion inhibited the expression of lipid related factors in ethanol/TGF-β-stimulated LX-2 cells

**FIGURE 7:**
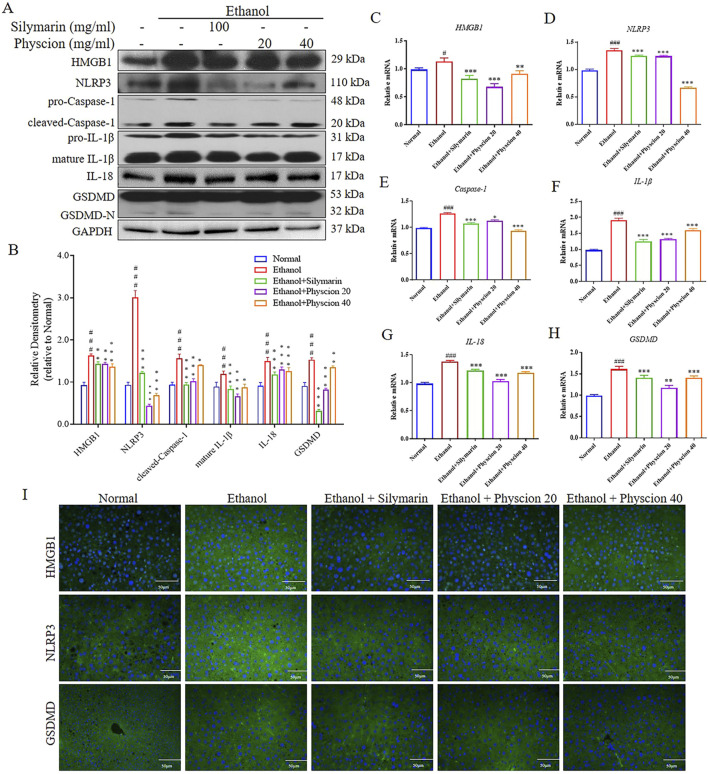
Physcion inhibited the expression of lipid related factors in ethanol/TGF-β-stimulated LX-2 cells. **(A–C)** The protein expression levels and relative protein expression statistics of Collagen-I and α-SMA; **(D, E)** The mRNA levels of Collagen-I and α-SMA. **(F)** Oil Red O staining of LX-2 cells (scale bars 50 µm); **(G, H)** The protein expression levels and relative protein expression statistics of SirT1, AMPKα, p-AMPKα, ACC, p-ACC and SREBP1; **(I)** The mRNA levels of SirT1. Significant differences between the indicated groups: ^##^ *P* < 0.01, ^###^ *P* < 0.001 vs. Control group; *P < 0.05, **P < 0.01, ***P < 0.001 vs. Ethanol/TGF-β group.

Upon liver injury or stimulation, HSCs are activated to differentiate into proliferative, contractile, inflammatory, and characterized by the synthesis of α-SMA and an increase in the ECM (Cai et al., 2020). In this experiment, ethanol combined with TGF-β induction model was used to stimulate LX-2 cells activation. The results showed that ethanol/TGF-β could activate LX-2 cells by increasing the protein and mRNA levels of the collagen deposition factor (Collagen-Ⅰ) and the marker of HSCs activation (α-SMA). After co-administration with physcion effectively ameliorated Collagen-Ⅰ and α-SMA expression (Figures 7A–E). In addition, we made use of GA (HMGB1 inhibitor) and MCC950 (NLRP3 inhibitor) to confirm HMGB1 and NLRP3 is effective on ethanol-induced LX-2 cells and physcion could inhibit the activation of HMGB1/NLRP3 inflammasome pathway. Importantly, GA and MCC950 reduced the protein and mRNA expression of these two factors as expected (Figures 7A–E).

Lipid metabolism is regulated by a variety of transcription factors, SREBP1 regulates the fatty acid and TG synthesis. Thus, regulation of physcion on the formation of lipid droplets in LX-2 cells can reflect the effect of physcion on lipid accumulation. As shown in Figure 7F, ethanol exposure increased lipid accumulation while physcion treatment reduced the deposition of lipid droplets in hepatocytes. As expected, the levels of SREBP1 protein were significantly elevated and SirT1, phosphorylation of AMPKα and ACC were significantly inhibited in LX-2 cells. Co-treatment with physcion restored the alteration, which was similar to the results in the GA and MCC950 (Figures 7G, H). At the mRNA level, we validated SirT1 gene in LX-2 cells, which was consistent with protein expression results (Figure 7I). These results indicated that physcion treatment could regulate the disturbance of HSCs lipid metabolism after ethanol/TGF-β challenge.

### Physcion mediated hepatocyte protection via HMGB1 pathways and pyroptosis in ethanol/TGF-β-stimulated LX-2 cells

**FIGURE 8:**
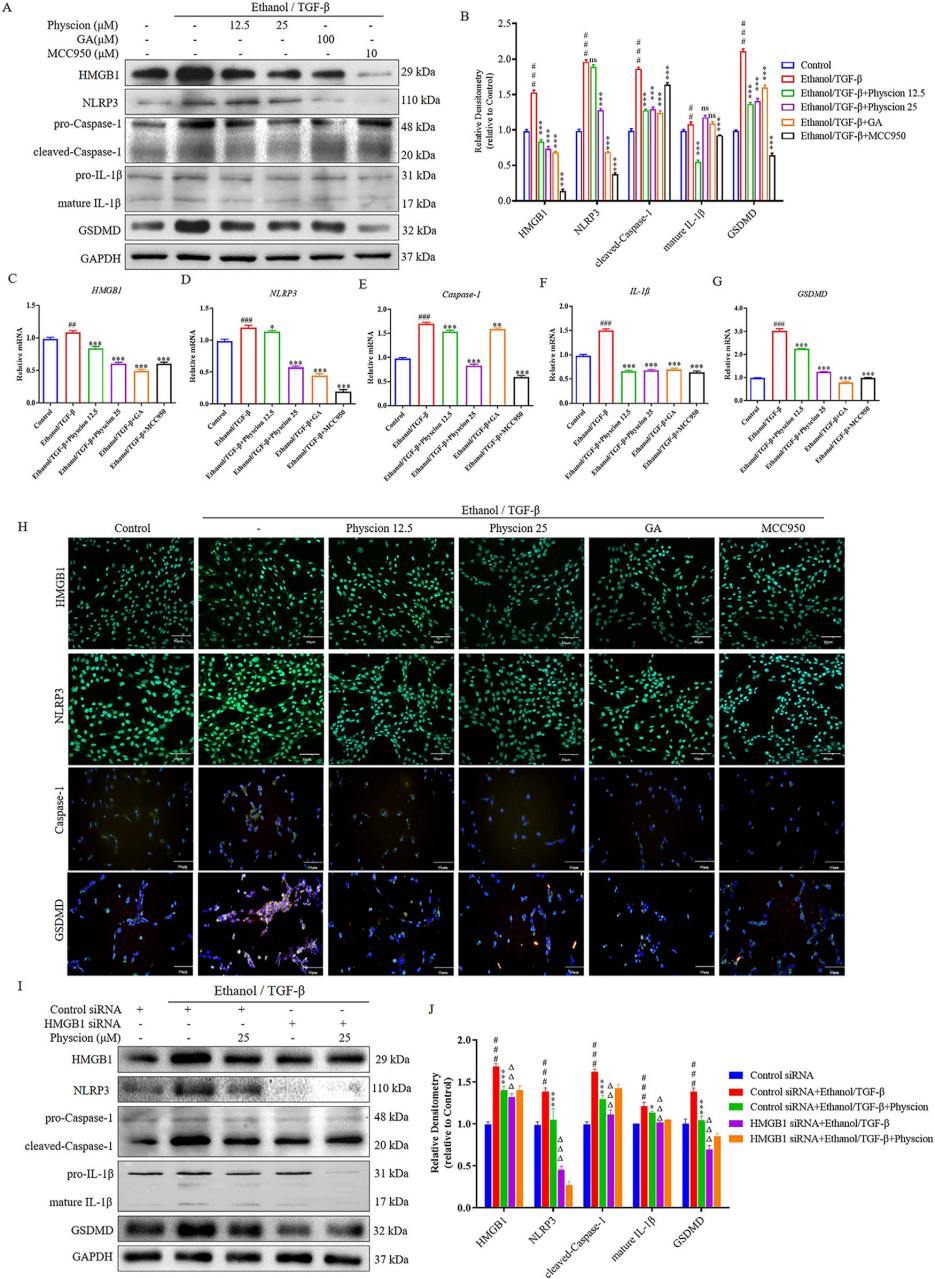
Physcion mediated hepatocyte protection via HMGB1 pathways and pyroptosis in ethanol/TGF-β-stimulated LX-2 cells. **(A, B)** The protein expression levels and relative protein expression statistics of HMGB1, NLRP3, cleaved-Caspase-1, mature IL-1β and GSDMD; **(C–G)** The mRNA levels of HMGB1, NLRP3, Caspase-1, IL-1β and GSDMD; **(H)** Immunofluorescence staining of HMGB1, NLRP3, Caspase-1 and GSDMD in LX-2 cells (scale bars 50 µm); **(I, J)** After pretreatment with HMGB1 siRNA, protein levels of HMGB1, NLRP3, cleaved-Caspase-1, mature IL-1β and GSDMD in ethanol/TGF-β-stimulated LX-2 cells were analyzed and their protein bands were normalized statistically. Significant differences between the indicated groups: ^##^ *P* < 0.01, ^###^ *P* < 0.001 vs. Control or Control siRNA group; *P < 0.05, **P < 0.01, ***P < 0.001 vs. Ethanol/TGF-β group; ^ΔΔΔ^ *P* < 0.001 vs. Ethanol/TGF-β + HMGB1 siRNA group; NS: not significant.

Hepatocyte pyroptosis and the release of inflammasome particles caused by DAMPs and PAMPs further induced HSCs activation and liver fibrosis (Wree et al., 2014). In order to explore physcion works by HMGB1 pathways and pyroptosis, we detected HMGB1 and pyroptosis-related factors. As shown in Figures 8A–G, with the ethanol/TGF-β-stimulated LX-2 cells for 6 h, HMGB1, NLRP3, Caspase-1, IL-1β and GSDMD proteins and mRNA levels were gradually increased, whereas these alterations were significantly alleviated after the intervention with physcion, GA and MCC950. In immunofluorescence, physcion significantly suppressed HMGB1, NLRP3, Caspase-1 and GSDMD expressions (Figure 8H). The above results suggested that HMGB1 pathways and Caspsse-1 dependent pyroptosis partially contributed to physcion induced hepato-protective effects against ALF.

To confirm that HMGB1 works in LX-2 cells, HMGB1 siRNA was used to knockdown HMGB1 gene expression. When LX-2 cells were transfected with HMGB1 siRNA for 48 h, the level of HMGB1 protein was markedly reduced in ethanol/TGF-β-stimulated LX-2 cells. HMGB1 knockdown inhibited ethanol/TGF-β induced inflammatory factors NLRP3, Caspase-1, IL-1β and pyroptosis-related factors GSDMD expression. After pretreatment of LX-2 cells with HMGB1-siRNA, the relief effect of physcion on pyroptosis of LX-2 cells was not further enhanced (Figures 8I, J). These results are consistent with physcion in ethanol/TGF-β-stimulated LX-2 cells, indicating that HMGB1 may participate in the inflammatory response and physcion suppresses the occurrence of pyroptosis by inhibiting HMGB1/NLRP3/GSDMD signaling pathway, which contributed to physcion’s hepatocyte protection.

### Physcion suppressed HMGB1-induced inflammatory response in Raw264.7 cells

**FIGURE 9:**
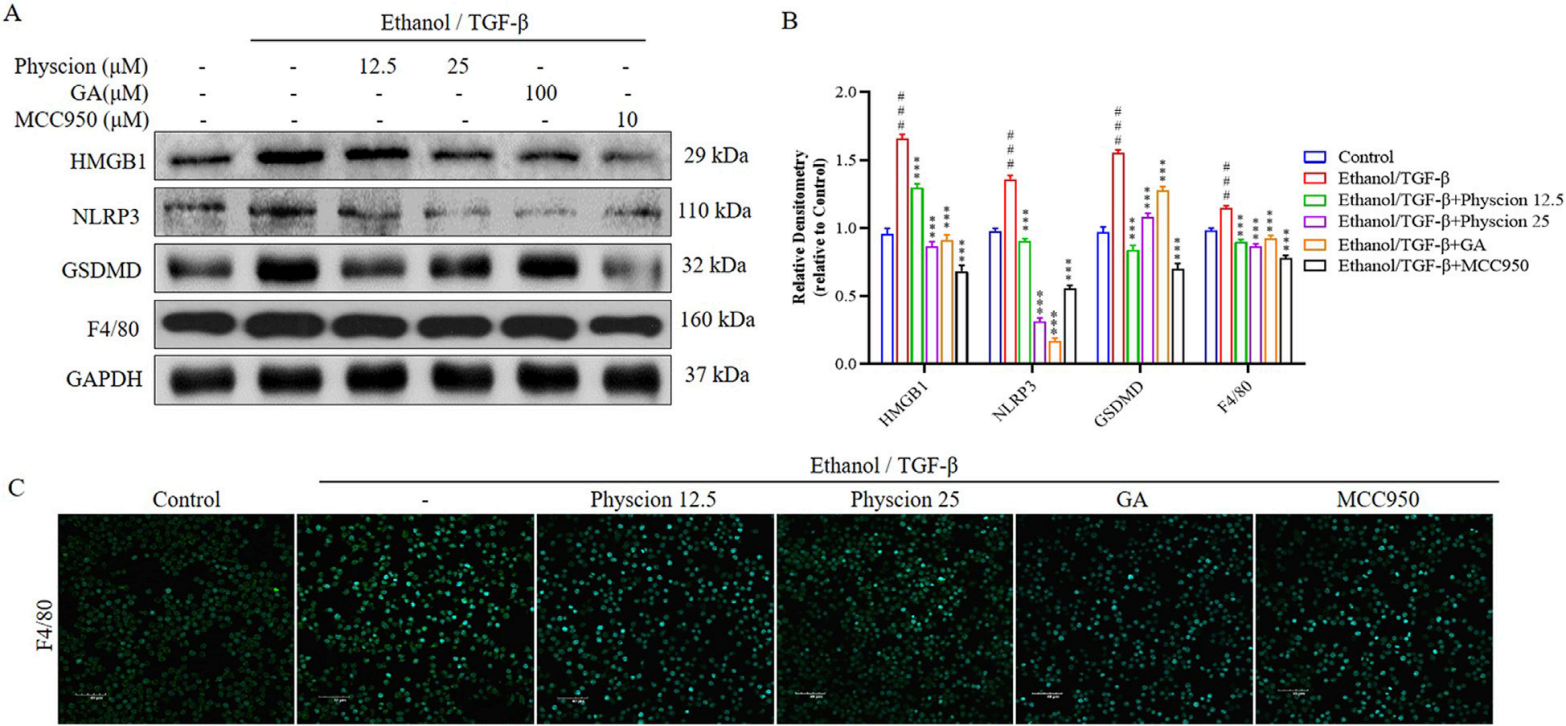
Physcion suppressed HMGB1-induced inflammatory response in Raw264.7 cells. **(A, B)** The protein expression levels and relative protein expression statistics of HMGB1, NLRP3, GSDMD and F4/80; **(C)** Immunofluorescence staining of F4/80 in Raw264.7 cells (scale bars 40 µm). Significant differences between the indicated groups: ^###^ *P* < 0.001 vs. Control group; ***P < 0.001 vs. Ethanol/TGF-β group.

Inflammasome activation and the subsequent pyroptosis in macrophages promote chronic liver inflammation, which initiate HSCs-mediated fibrosis from various etiologies (Tacke, 2017). We performed the same validation with RAW264.7 cells, the expression of HMGB1, NLRP3, GSDMD and F4/80 (a marker of macrophages) proteins was significantly increased in the ethanol/TGF-β-stimulated group, and the physcion groups had varying degrees of inhibition of the expression of these proteins, which was similar to that in the GA and MCC950 groups (Figures 9A, B). In addition, we measured the effect of physcion on the expression of F4/80 protein in RAW264.7 cells by immunofluorescence. The expression of F4/80 after ethanol/TGF-β challenge were significantly higher in the control group, and significantly decreased in the physcion, GA and MCC950 groups (Figure 9C). Overall, our data indicate that physcion alleviate ALF by inflammasome activation mediates the liver macrophages and subsequent interaction with HSCs, contributing to the progression of liver fibrosis.

## Discussion

**FIGURE 10:**
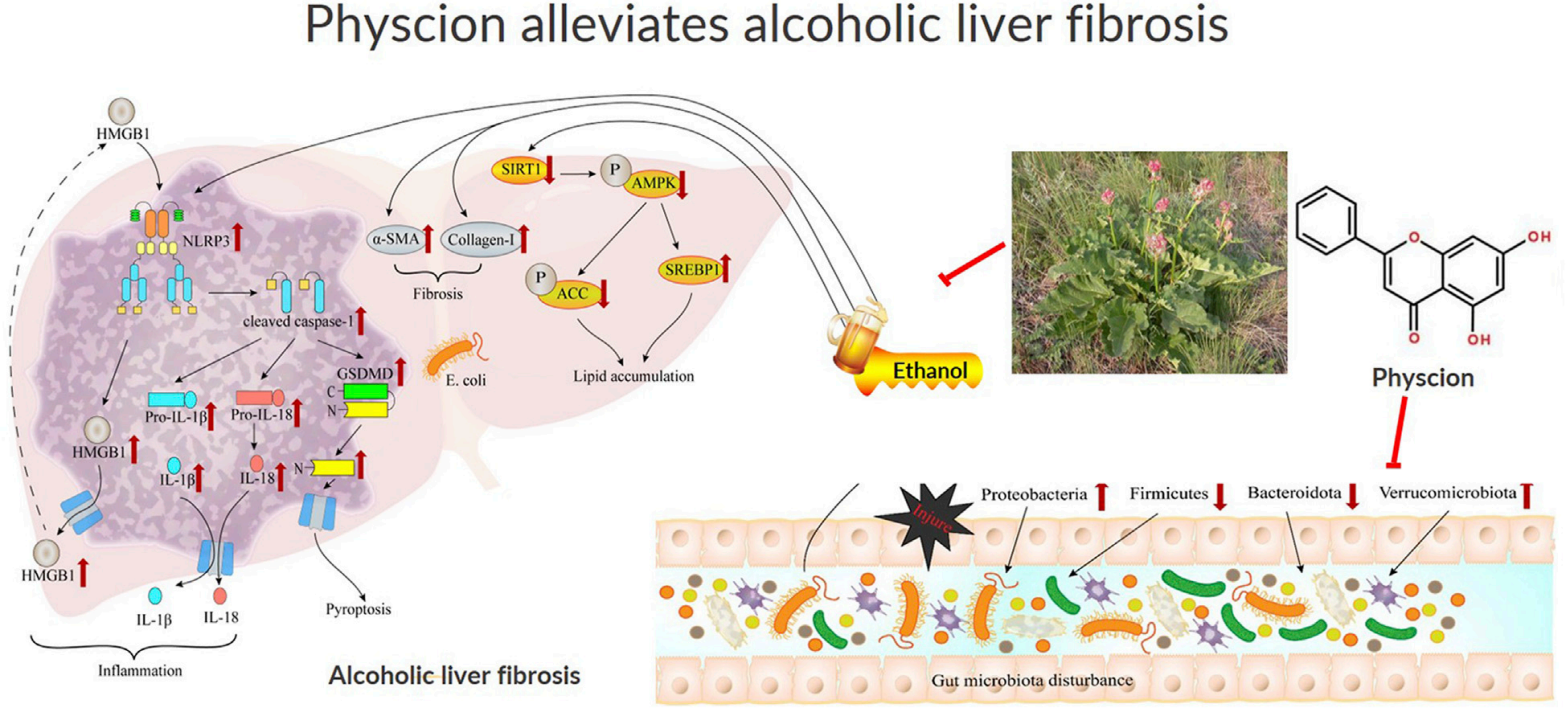
Schematic illustration of the effect of physcion in ameliorating ALF and the pathway of HMGB1/NLRP3 and gut microbiota in ALF development. Physcion activated SirT1/AMPK and inhibited generation of pro-inflammatory molecules including IL-1β, IL-18 and Caspsse-1 dependent pyroptosis in the liver, which alleviate chronic liver injury, steatosis and inflammation induced by chronic-binge ethanol consumption. Further mechanistic study suggests that the protective effects of physcion administration is mediated through suppressing the activation of HMGB1/NLRP3/GSDMD both in the cells and liver. Physcion treatment alleviated alcohol-induced gut microbiota disorders by inhibiting *Escherichia coli* in liver and restoring the diversity of intestinal flora. Finally, physcion administration attenuates gut microbiota and HMGB1/NLRP3 inflammasome pathways in the chronic-binge ethanol consumption induced ALF mouse model.

Physcion is a cost-effective agent, enriched in natural plants and marine resources. It is known that physcion exerts a therapeutic role in a variety of diseases. Physcion inhibit tumor growth and metastasis in various cancer models, including breast, lung, and liver cancers, by inducing apoptosis, cell cycle arrest, and inhibiting angiogenesis (Trybus et al., 2021). Its anti-inflammatory properties make it a potential candidate for treating chronic inflammatory diseases, such as rheumatoid arthritis and inflammatory bowel disease. Additionally, its antioxidant activity suggests potential applications in neurodegenerative diseases (Li et al., 2023). Although many basic and clinical studies have been conducted on Rhubarb, there has been no report until now evidencing the effect of physcion on ethanol-induced ALF. In this study, we found that physcion could alleviate ethanol induced liver fibrosis, and proposed that the beneficial effect of physcion on ALF may be achieved through the HMGB1/NLRP3/GSDMD signaling pathway and improving gut microbiota (Figure 10).

ALF is a chronic progressive disease, which is an advanced reversible stage in the development of ALD (Seitz et al., 2018). There is growing evidence that ALF is reversible, with hepatocyte damage and HSCs being activated after alcohol consumption, which leads to excessive accumulation of ECM and acetaldehyde metabolized in the ethanol liver. On the other hand, the mechanism of acetaldehyde-induced fibrosis relies on the TGF-β pathway (Hyun et al., 2021). In the process of liver fibrosis, there are cytokines, signaling pathways, and various cellular interactions. This is often accompanied by hepatic steatosis and inflammation, with varying degrees of increased expression of inflammasomes in macrophages, HSCs, and hepatocytes (Yang et al., 2022). Once the liver is injury, it is inevitably accompanied by a robust inflammatory response, with damaged or dead hepatocytes transmitting inflammatory signals through DAMPs and the release of cytokines such as IL-1β further enhances the activation of HSCs (Gaul et al., 2021). In this study, it was found that alcohol stimulation could upregulate the inflammatory factor NLRP3, Caspase-1 and IL-1β levels, further prompt the synthesis of downstream factor Collagen-Ⅰ and α-SMA, and significantly alleviate this phenomenon after physcion treatment, and the effect was similar to that of silymarin.

The earliest reaction to alcoholism is the accumulation of lipids in liver cells. AMPK inhibits SREBP1 activity, which regulates protein cleavage maturation and intranuclear translocation, thereby inhibiting the expression of lipid-stimulating target protein fatty acid synthase (Anggreini et al., 2023). SirT1 is a protein that regulates metabolism, including adipocyte accumulation and maturation, hepatic lipid metabolism, and systemic inflammation (Anggreini et al., 2023). Results suggest that SirT1 and AMPK play an important role in the progression of lipid-related diseases, including ALF (He L. et al., 2023). More importantly, SirT1 regulates SREBP1 activity, resulting in transcriptional repression of the SREBP1-targeted lipogenic enzymes such as FAS, SCD, and ACC in cultured hepatocytes and mouse liver. Our results suggest that SirT1 has a certain regulatory effect on ALF mice. In ethanol/TGF-β-stimulated LX-2 cells, AMPK phosphorylation decreased and SREBP1 expression increased. After physcion intervention, the form was reversed. Above all, physcion remarkably alleviated SirT1, AMPK and SREBP1 mediated liver steatosis in the mice exposed with chronic-binge ethanol consumption.

HMGB1, a nuclear DNA-binding protein that stabilizes nucleosomes and regulates gene expression, becomes a DAMP when it occurs in the extracellular environment and is perceived by the immune system as an endogenous danger signal, leading to the development of inflammatory diseases (Yang et al., 2020). Previous studies found that extracellular HMGB1 downregulation inhibit the activation of macrophage NLRP3 inflammasomes and hepatocyte HMGB1 secretion in an autophagy-dependent manner, thereby reducing extracellular HMGB1 and improving liver injury and fibrosis (He M. et al., 2023). This is consistent with our findings, HMGB1 siRNA and HMGB1 inhibitor impeded alcohol-induced inflammatory factors IL-1β, Caspase-1 and NLRP3, importantly, physcion obviously suppresses the expression of HMGB1 in alcohol-induced mice and LX-2 cells.

Considering that the activation of HMGB1 is closely related to pyroptosis, we further investigated whether pyroptosis occurs in the liver with ALF. Pyroptosis is a programmed mode of cell death associated with an inflammatory response, consisting of three types: the Caspase-1-mediated classical pathway, the Caspase-4/5/11-mediated non-canonical pathway, and the Caspase-3 pathway (Jin et al., 2023). The formation of inflammasomes converts the pro-Caspase-1 precursor to activated Caspase-1, and the activation of Caspase-1 promotes the cleavage of inactive IL-1β and IL-18 precursors to produce mature IL-1β and IL-18 (Al Mamun et al., 2020). In addition, activated Caspase-1 acts on GSDMD, cleaving GSDMD to produce reactive amino (N) and carboxyl (C) terminus. The interterminal domain is lipid-selective and binds to phosphoinositide unique to eukaryotic cell membranes and cardiolipin unique to prokaryotic cells, resulting in 10–20 nm pores in cell membranes, through which small molecules IL-1β and IL-18 are secreted extracellularly, ultimately leading to pyroptosis (Shi et al., 2015). At the same time, HMGB1, which should normally exist in the nucleus, is stimulated by alcohol and metastasis from the nucleus to the cytoplasm, and the cell membrane integrity is lost due to pyroptosis, which makes HMGB1 more easily released outside the cell to trigger inflammatory responses, forming a vicious circle.

Liver fibrosis is a slow process that requires the involvement of various cells and cytokines. The liver is composed of nonparenchymal cells (HSCs, kupffer cells, and immune cells) and hepatocytes (Kisseleva and Brenner, 2021). In persistent liver injury, many hepatocytes die in pyroptosis, followed by the release of intracellular substances, and these paracrine molecules act as endogenous signals that can activate inflammasomes in HSCs, further promoting the secretion of cytokines, which in turn leads to collagen deposition and liver fibrosis (Yang et al., 2022). So far, there are few studies on the effect of HSCs pyroptosis on liver fibrosis. Therefore, we explored the effect of physcion on pyroptosis-related cytokines during ALF, and the results showed that at the protein and mRNA level, physcion could significantly reduce the expression of inflammasome-associated proteins NLRP3, Caspase-1, IL-1β and GSDMD, reduce hepatocyte pyroptosis, and alleviate the progression of ALF.

Intestinal flora is associated with ALD and is involved in pathological processes such as hepatocellular steatosis, alcoholic hepatitis, ALF, and liver cirrhosis (Wang et al., 2021). The connection between the liver and the gut is achieved through the gut-liver axis, genetic, nutritional, and environmental variables all influence the interaction between the two (Zhao et al., 2022). In recent years, attention has been focused on the gut microbiota (Zhang et al., 2021). In ALF, the interaction between the microbiome and the host liver is of particular concern, and there has been evidence that alcohol alters the composition of the gut microbiota and impairs gut integrity and barrier function. On the one hand, alcohol can cause damage and death of intestinal epithelial cells (Wrzosek et al., 2021). The alcohol metabolite acetaldehyde disrupts tight junctions, resulting in increased intestinal permeability. On the other hand, long-term alcohol consumption increases LPS levels in the portal vein and systemic circulation. LPS receptor binding activates Kuffer cells and macrophages, the latter activating the nuclear factor kappa-B (NF-κB) pathway. This pathway induces the production of inflammatory chemokines, including TNF-α and IL-1β (Liu et al., 2022). Our results showed that the richness of intestinal microbiota and species diversity of drinking mice were significantly reduced. Taken together, changes in the gut microbiota contribute to ALD via different mechanisms, which include disruption of the intestinal barrier, secretion of toxins, and metabolism of microbial molecules. Alcohol-associated dysbiosis represents an attractive target to regulate ethanol-induced liver inflammation and hepatocyte damage.

Our study showed that the intestinal bacteria changed during ALF, and this change was closely associated with physcion-mediated inhibition of the HMGB1/NLRP3 inflammasome pathway. Short-chain fatty acids (SCFAs), such as acetate, propionate, and butyrate, produced by the fermentation of dietary fibers by intestinal bacteria, have been shown to inhibit the HMGB1/NLRP3 pathway. Recent studies have demonstrated that SCFAs-driven NLRP3 inflammasome activation in macrophages (Wang et al., 2024). Additionally, SCFAs enhance intestinal epithelial barrier function, thereby reducing HMGB1 release and systemic inflammation (Kim et al., 2013). The intestinal bacteria also play a critical role in maintaining the integrity of the gut barrier, which is essential for preventing the leakage of luminal contents into the systemic circulation and triggering inflammation. Disruptions in gut barrier function, such as those caused by intestinal infections or inflammation, can lead to increased permeability and the release of DAMPs like HMGB1 (de Vos et al., 2022). After physcion treatment, the number of *E. coli* in the liver tissue of mice was significantly reduced, indicating that physcion promoted the recovery of intestinal barrier function. In addition, 16S rRNA sequencing data revealed aberrant microbial composition at different taxa levels. At the genus level, physcion effectively reversed the increase of harmful bacteria, such as *Escherichia-Shigella* and *Akkermansia* caused by alcohol consumption, and at the phylum level, physcion promoted the recovery of beneficial bacteria *Firmicutes* microorganisms in the intestinal tract of mice. These results provide a reference for exploring the role of physcion in the treatment of liver inflammation, liver fibrosis and the signal transduction pathway of intestinal flora. While preclinical studies highlight its efficacy in diseases such as cancer and rheumatoid arthritis, its clinical translation faces challenges, including dose-dependent nephrotoxicity, moderate bioavailability, but its ability to target multiple pathways simultaneously makes it a promising candidate for combination therapy, particularly in liver injury treatment. Furthermore, its natural origin and relatively low cost of extraction make it an attractive option for further development.

## Conclusion

In conclusion, physcion can improve liver steatosis, hepatocyte pyroptosis, liver fibrosis and intestinal flora disturbance in ALF mice, and play a protective role in ALD. The mechanism may be to alleviate hepatocyte pyroptosis by inhibiting the HMGB1/NLRP3/GSDMD signaling pathway, thus slowing down the alcohol-induced liver fibrosis process. This study provides new evidence to elucidate the mechanism of physcion on ALF, and provides new insights for the prevention and treatment of chronic liver disease.

## Data Availability

The data presented in the study are deposited in the NCBI SRA database, accession number PRJNA1241684.

## Abbreviations

α-SMA, α-smooth muscle actin; AKP, alkaline phosphatase; ALD, alcohol-associated liver disease; ALF, alcoholic liver fibrosis; ALT, alanine aminotransferase; AMPK, adenosine monophosphate-activated protein kinase; AST, aspartate aminotransferase; Caspase-1, cysteinyl aspartate specific proteinase-1; DAMPs, damage-associated molecular patterns; GSDMD, gasdermin D; HMGB1, high mobility group box 1 protein; HSCs, hepatic stellate cells; IL-1β, interleukin-1β; NLRP3, NOD-like receptor protein 3; PAMPs, pathogen-associated molecular patterns; SirT1, sirtuin 1; SREBP1, sterol regulatory element-binding protein 1; TG, triglyceride; TGF-β, transforming growth factor-β; TNF-α, tumor necrosis factor-α.
