# Supplementary material for: Gut microbiota and HMGB1/NLRP3/GSDMD inflammasome-dependent pyroptosis: mechanisms by physcion ameliorates alcoholic liver fibrosis
Source: Front Pharmacol. 2025 Mar 27;16:1532590. doi: 10.3389/fphar.2025.1532590 (PMC11982826; doi:10.3389/fphar.2025.1532590)
Supplement: Supplementary file 1 [file Table1.pdf]

Supplementary Table S1. Primers sequences used in RT-PCR.

Gene	Accession No.	Species	Primer Sequence
Collagen- Ⅰ	NM_007742.3	*Mus musculus*	5′-TGAGTCAGCAGATTGAGAAC-3′
			5′-TACTCGAACGGGAATCCATC-3′
α-SMA	NM_007392.2	*Mus musculus*	5′-CATCAGGGAGTAATGGTTGG-3′
			5′-CACAATACCAGTTGTACGTC-3′
SirT1	NM_001159589.2	*Mus musculus*	5′-GACGCTGTGGCAGATTGTTA-3′
			5′-GGAATCCCACAGGAGACAGA-3′
SREBP1	XM_006532716.4	*Mus musculus*	5′-AGGTGTATTTGCTGGCTTGGT-3′
			5′-AGAGATGACTAGGGAACTGTGTGT-3′
HMGB1	NM_010439.4	*Mus musculus*	5′-GCCCATTTTGGGTCACATGG-3′
			5′-TGCAGGGTGTGTGGACAAAA-3′
NLRP3	XM_036156549.1	*Mus musculus*	5′-ATCAACAGGCGAGACCTCTG-3′
			5′-GTCCTCCTGGCATACCATAGA-3′
Caspase-1	NM_009807.2	*Mus musculus*	5′-ACAAGGCACGGGACCTATG-3′
			5′-TCCCAGTCAGTCCTGGAAATG-3′
IL-1β	NM_008361.3	*Mus musculus*	5′-GTACATCAGCACCTCACAAG-3′
			5′-CACAGGCTCTCTTTGAACAG-3′
IL-18	NM_008360.2	*Mus musculus*	5′-TGACCCTCTCTGTGAAGGATAG-3′
			5′-TTTCAGGTGGATCCATTTCCTC-3′
GSDMD	NM_026960.4	*Mus musculus*	5′-CCATCGGCCTTTGAGAAAGTG-3′
			5′-ACACATGAATAACGGGGTTTCC-3′
GAPDH	NM_008084.2	*Mus musculus*	5′-CTTGTGCAGTGCCAGCC-3′
			5′-GCCCAATACGGCCAAATCC-3′
Collagen- Ⅰ	NM_000088.3	*Homo sapiens*	5′-CAAGACGAAGACATCCCAC-3′
			5′-CGGTTGATTTCTCATCATAGC-3′
α-SMA	NM_001141945.1	*Homo sapiens*	5′-TCGTTACTACTGCTGAGCGTG-3′
			5′-TCTCCTTCTGCATTCGGTCG-3′
SirT1	NM_012238.5	*Homo sapiens*	5′-TGCCGGAAACAATACCTCCA-3′
			5′-AGACACCCCAGCTCCAGTTA-3′
HMGB1	NM_001363661.2	*Homo sapiens*	5′-TCAAAGGAGAACATCCTGGCCTGT-3′
			5′-CTGCTTGTCATCTGCAGCAGTGTT-3′
NLRP3	NM_004895.4	*Homo sapiens*	5′-GGTGTTGGAATTAGACAACTGC-3′
			5′-TCAAAGACGACGGTCAGCTC-3′
Caspase-1	NM_033292.3	*Homo sapiens*	5′-ACATCCTTCATCCTCAGAAAC-3′
			5′-GATAATGAGGGCAAGACGTG-3′
IL-1β	NM_000576.2	*Homo sapiens*	5′-CCTGTACGATCACTGAACTG-3′
			5′-TTGGGATCTACACTCTCCAG-3′
GSDMD	NM_024736.7	*Homo sapiens*	5′-AGACCATCTCCAAGGAACTG-3′
			5′-GGACAACACCAGGCACTC-3′
GAPDH	NM_002046.4	*Homo sapiens*	5′-GGCTCTCCAGAACATCATC-3′
			5′-CTCTTCCTCTTGTGCTCTTG-3′
