# Supplementary figures and images for: Gut microbiota and HMGB1/NLRP3/GSDMD inflammasome-dependent pyroptosis: mechanisms by physcion ameliorates alcoholic liver fibrosis
Source: Front Pharmacol. 2025 Mar 27;16:1532590. doi: 10.3389/fphar.2025.1532590 (PMC11982826; doi:10.3389/fphar.2025.1532590)

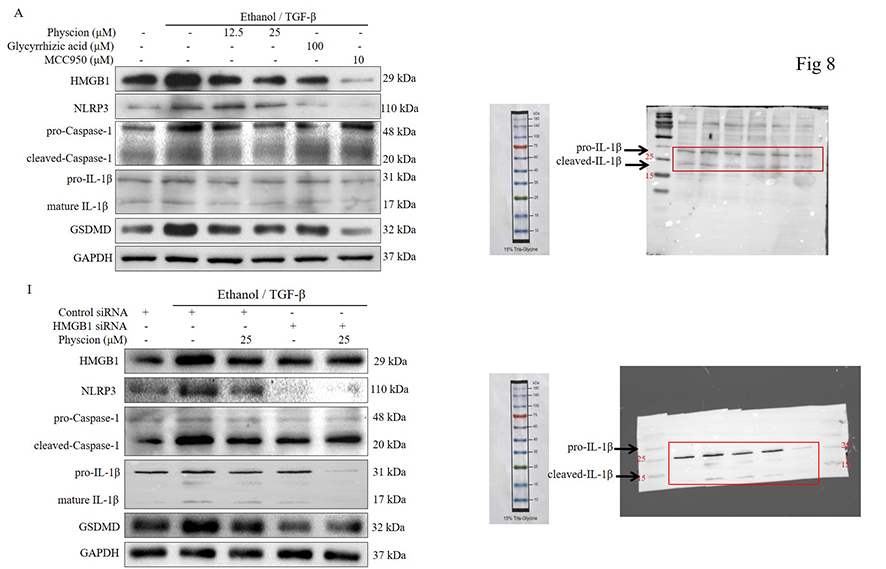

Supplement: Supplementary file 2 [file Image1.tif]
