# Supplementary material for: Gut microbiota and HMGB1/NLRP3/GSDMD inflammasome-dependent pyroptosis: mechanisms by physcion ameliorates alcoholic liver fibrosis
Source: Front Pharmacol. 2025 Mar 27;16:1532590. doi: 10.3389/fphar.2025.1532590 (PMC11982826; doi:10.3389/fphar.2025.1532590)
Supplement: Supplementary file 3 [file DataSheet1.pdf]

Fig 2

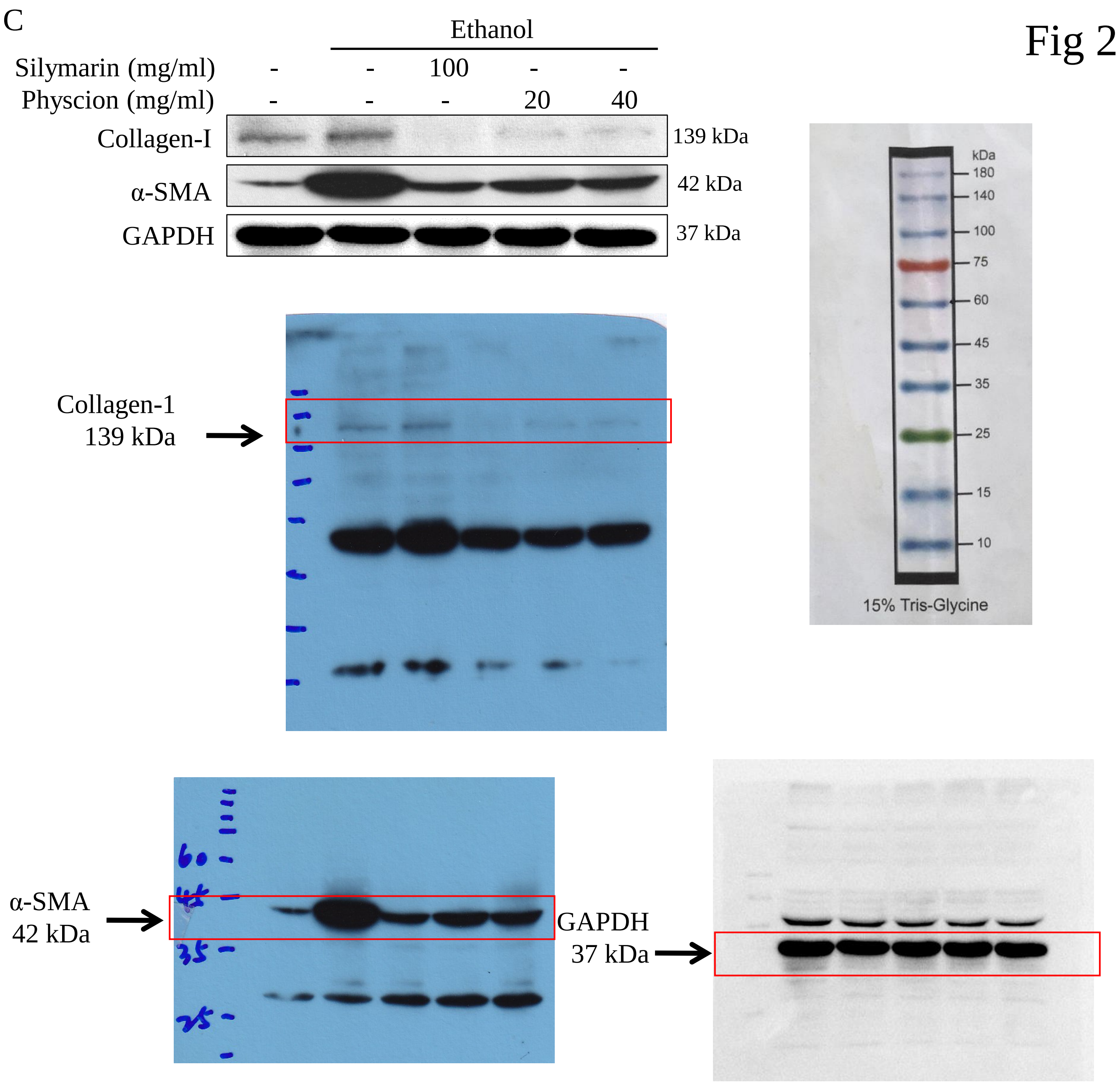
C
Ethanol
Silymarin (mg/ml) - - 100 - -
Physcion (mg/ml) - - - 20 40
Collagen-I 139 kDa
α-SMA 42 kDa
GAPDH 37 kDa
Collagen-1 139 kDa
kDa
180
140
100
75
60
45
35
25
15
10
15% Tris-Glycine
α-SMA 42 kDa
60
45
35
25
GAPDH 37 kDa

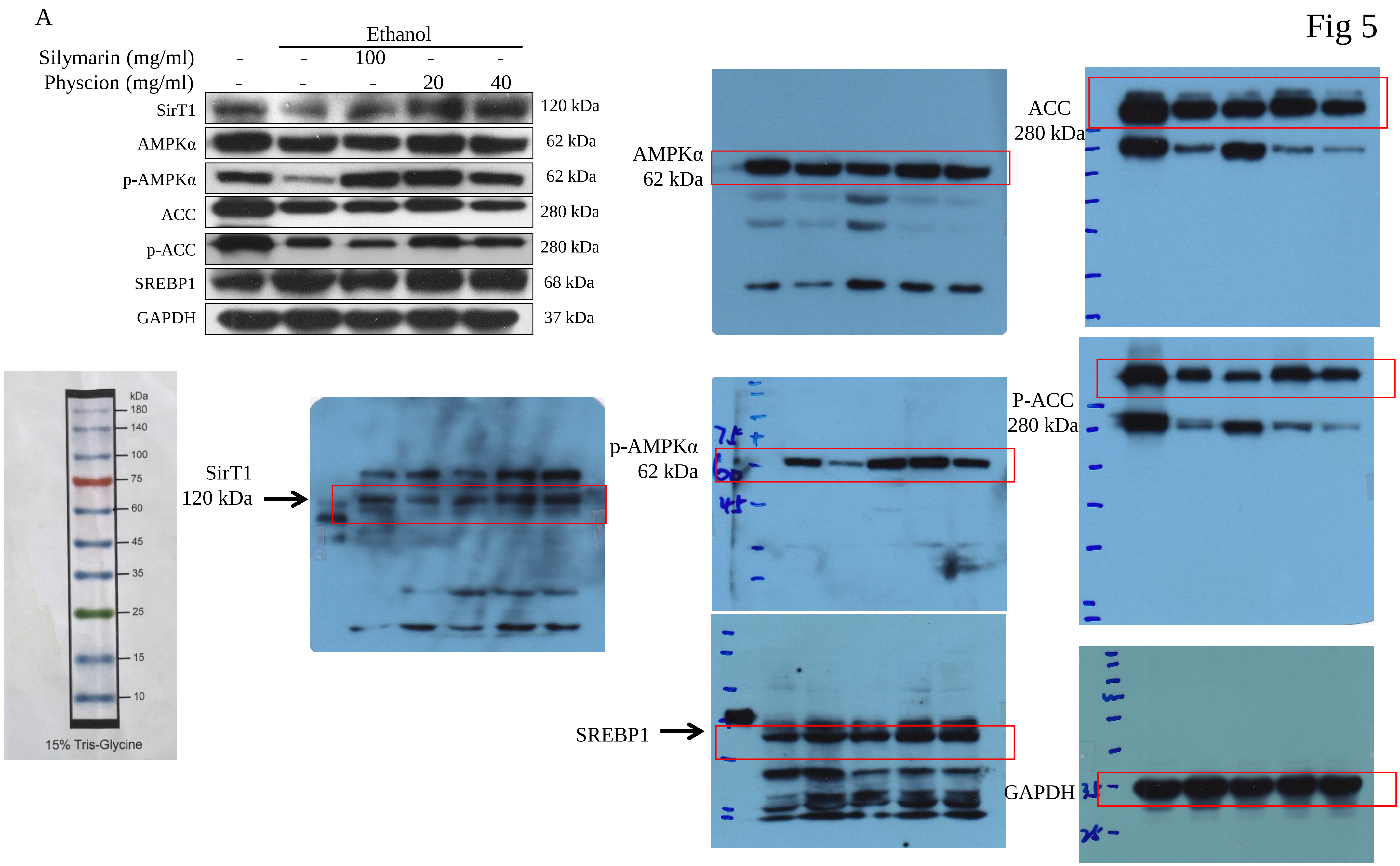

Fig 5
A
Ethanol
Silymarin (mg/ml) - - 100 - -
Physcion (mg/ml) - - - 20 40
SirT1 120 kDa
AMPKα 62 kDa
p-AMPKα 62 kDa
ACC 280 kDa
p-ACC 280 kDa
SREBP1 68 kDa
GAPDH 37 kDa
kDa
180
140
100
75
60
45
35
25
15
10
15% Tris-Glycine
SirT1 120 kDa
AMPKα 62 kDa
p-AMPKα 62 kDa
SREBP1
ACC 280 kDa
P-ACC 280 kDa
GAPDH

Fig 6

A

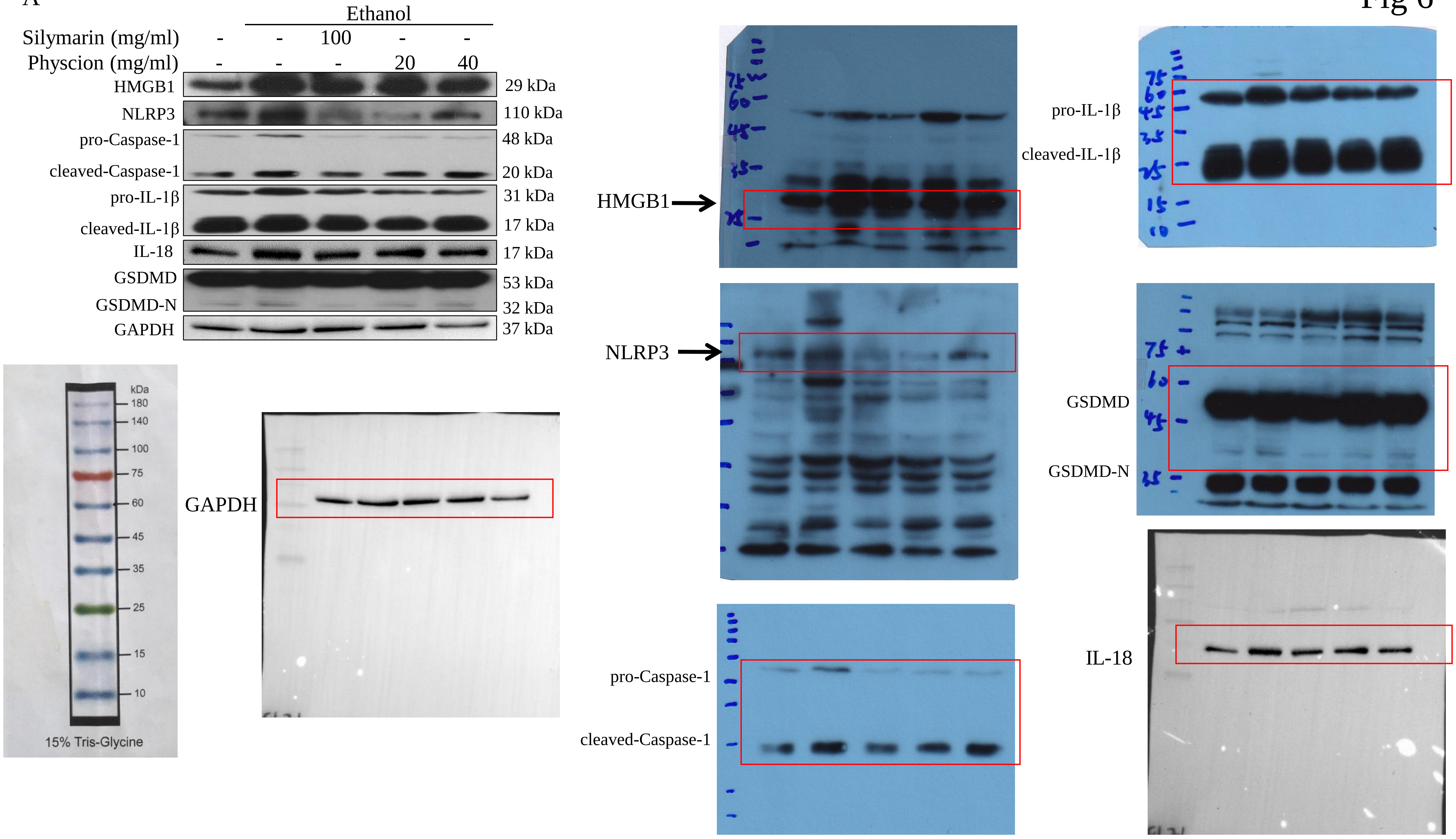
Ethanol
Silymarin (mg/ml) - - 100 - -
Physcion (mg/ml) - - - 20 40
HMGB1 29 kDa
NLRP3 110 kDa
pro-Caspase-1 48 kDa
cleaved-Caspase-1 20 kDa
pro-IL-1β 31 kDa
cleaved-IL-1β 17 kDa
IL-18 17 kDa
GSDMD 53 kDa
GSDMD-N 32 kDa
GAPDH 37 kDa
kDa
180
140
100
75
60
45
35
25
15
10
15% Tris-Glycine
GAPDH
HMGB1
NLRP3
pro-Caspase-1
cleaved-Caspase-1
pro-IL-1β
cleaved-IL-1β
GSDMD
GSDMD-N
IL-18

Fig 7

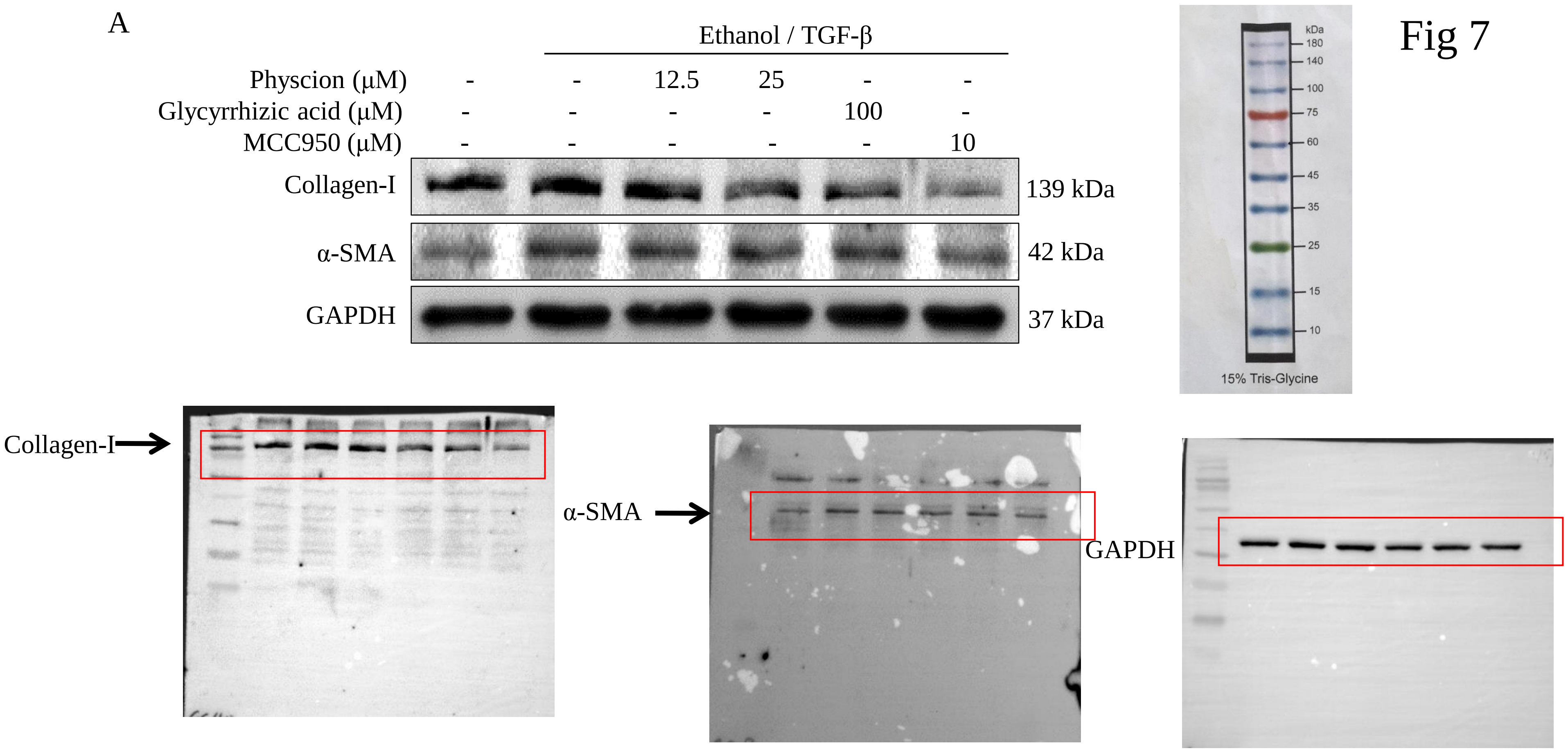
A
Ethanol / TGF-β
Physcion (μM) - - 12.5 25 - -
Glycyrrhizic acid (μM) - - - - 100 -
MCC950 (μM) - - - - - 10
Collagen-I
139 kDa
α-SMA
42 kDa
GAPDH
37 kDa
kDa
180
140
100
75
60
45
35
25
15
10
15% Tris-Glycine
Collagen-I
α-SMA
GAPDH

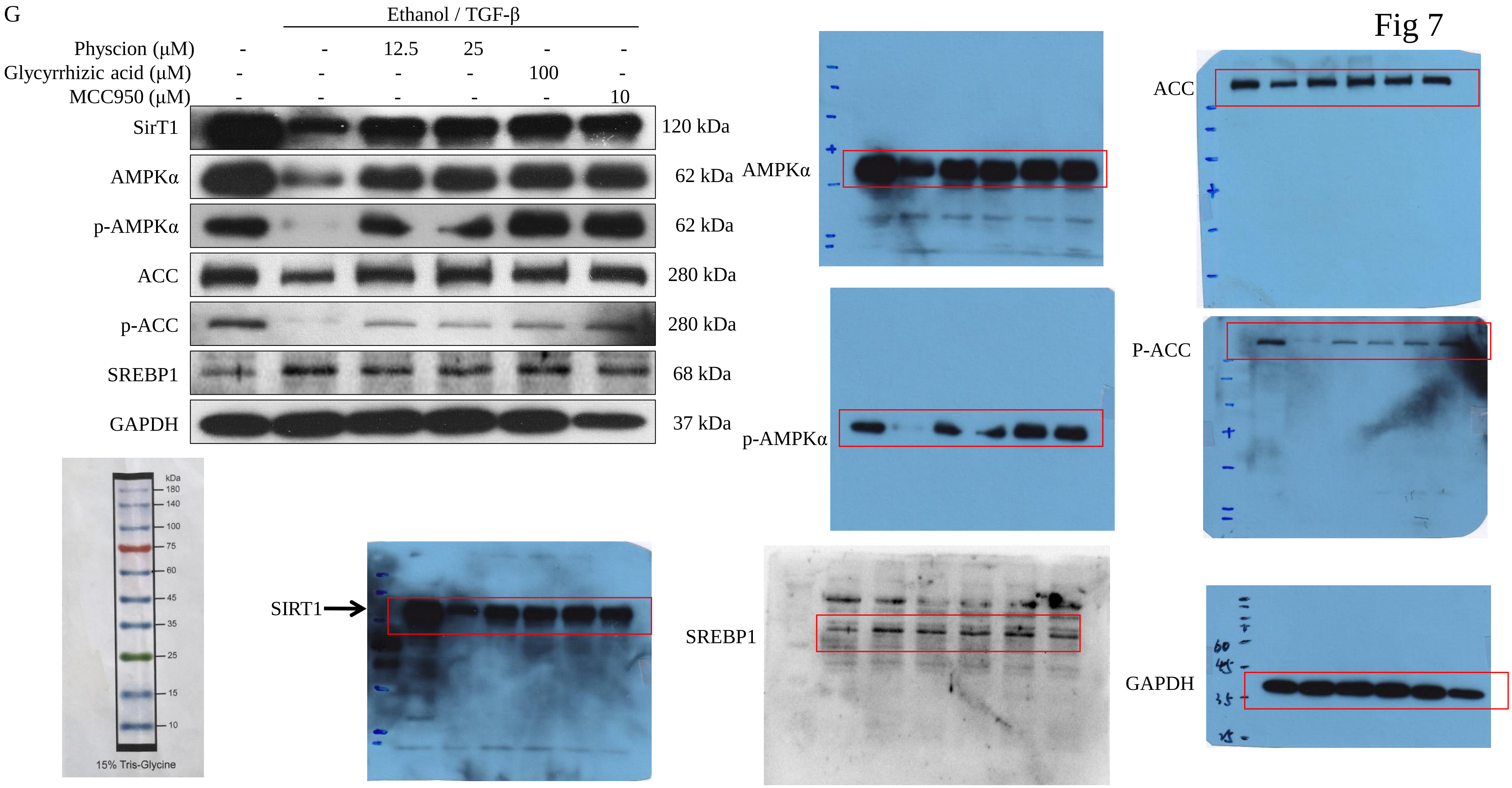
Fig 7
G
Ethanol / TGF-β
Physcion (μM) - - 12.5 25 - -
Glycyrrhizic acid (μM) - - - - 100 -
MCC950 (μM) - - - - - 10
SirT1 120 kDa
AMPKα 62 kDa
p-AMPKα 62 kDa
ACC 280 kDa
p-ACC 280 kDa
SREBP1 68 kDa
GAPDH 37 kDa
kDa
180
140
100
75
60
45
35
25
15
10
15% Tris-Glycine
SIRT1
AMPKα
p-AMPKα
SREBP1
ACC
P-ACC
GAPDH

Fig 8

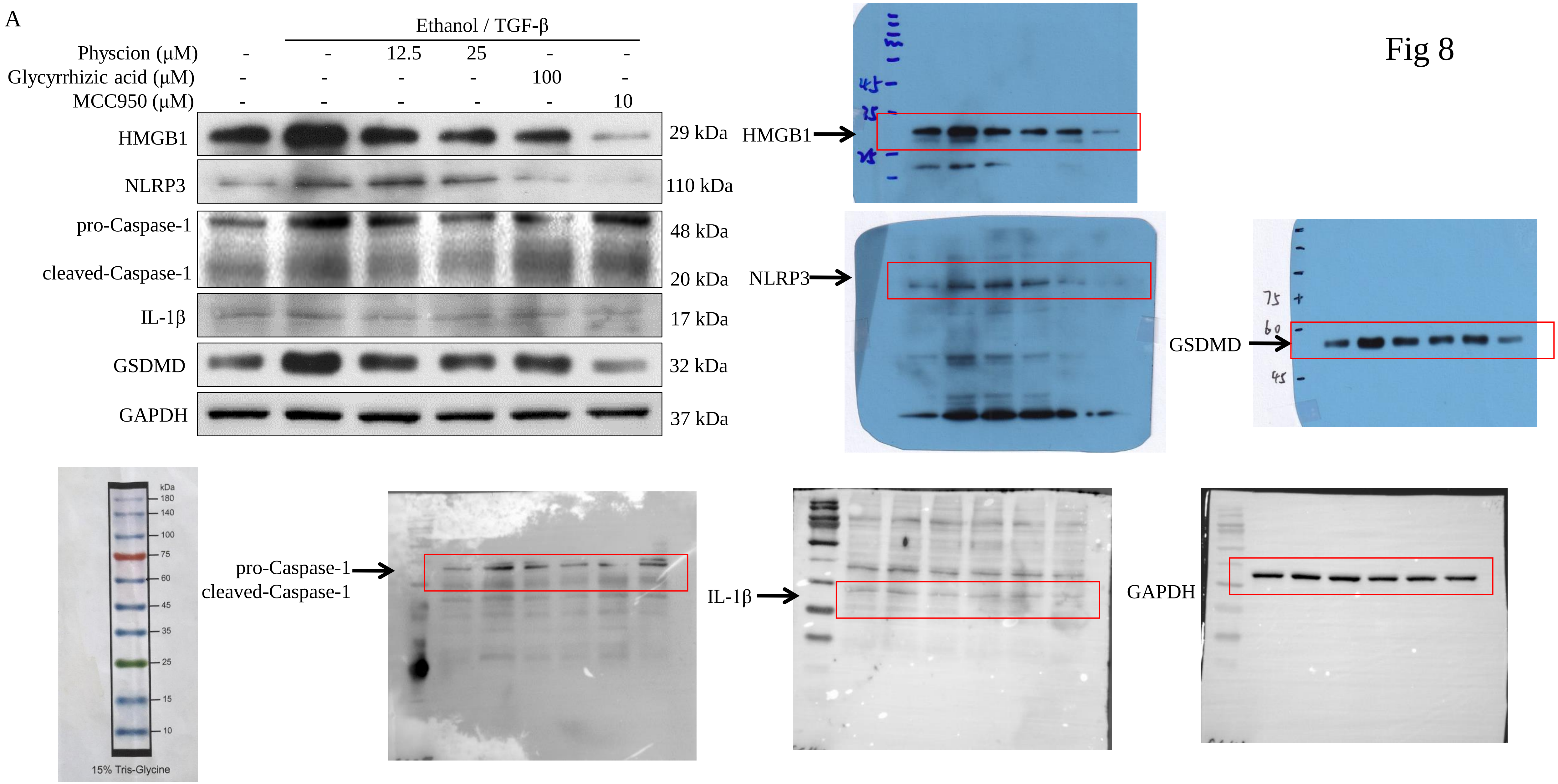
A
Ethanol / TGF-β
Physcion (μM) - - 12.5 25 - -
Glycyrrhizic acid (μM) - - - - 100 -
MCC950 (μM) - - - - - 10
HMGB1
29 kDa
NLRP3
110 kDa
pro-Caspase-1
48 kDa
cleaved-Caspase-1
20 kDa
IL-1β
17 kDa
GSDMD
32 kDa
GAPDH
37 kDa
HMGB1
NLRP3
GSDMD
kDa
180
140
100
75
60
45
35
25
15
10
15% Tris-Glycine
pro-Caspase-1
cleaved-Caspase-1
IL-1β
GAPDH

Fig 8

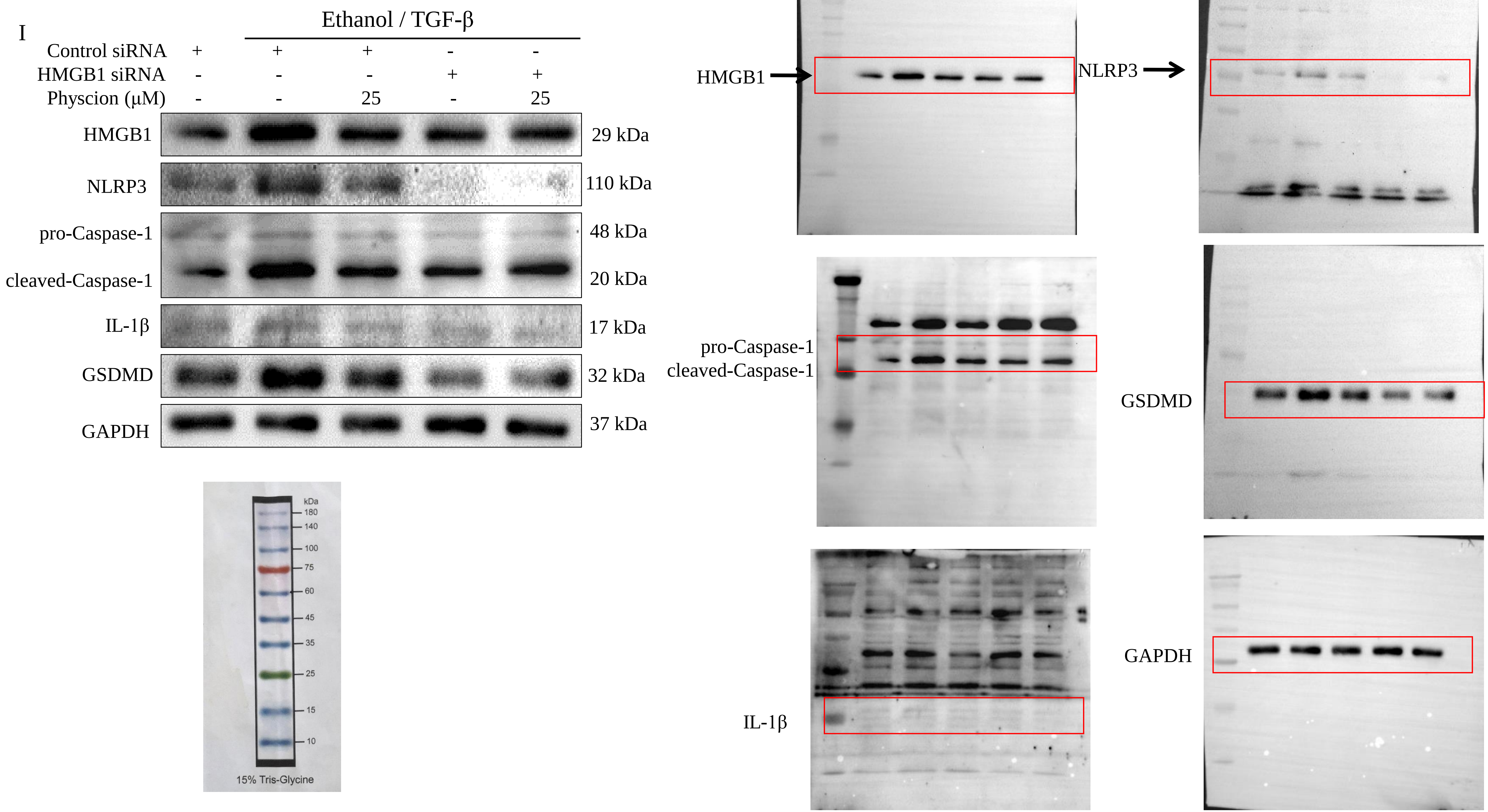
I
Ethanol / TGF-β
Control siRNA + + + - -
HMGB1 siRNA - - - + +
Physcion (μM) - - 25 - 25
HMGB1
29 kDa
NLRP3
110 kDa
pro-Caspase-1
48 kDa
cleaved-Caspase-1
20 kDa
IL-1β
17 kDa
GSDMD
32 kDa
GAPDH
37 kDa
kDa
180
140
100
75
60
45
35
25
15
10
15% Tris-Glycine
HMGB1
NLRP3
pro-Caspase-1
cleaved-Caspase-1
GSDMD
IL-1β
GAPDH

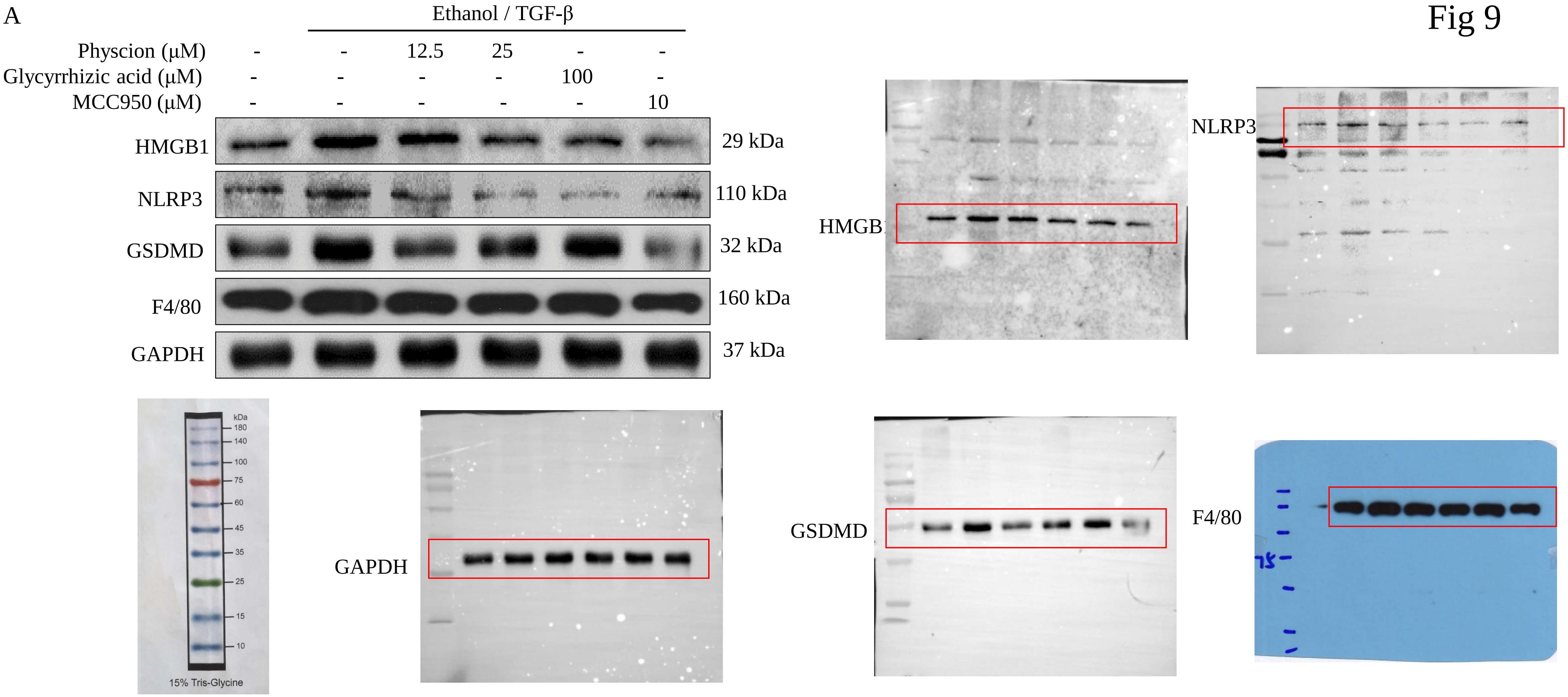
A
Ethanol / TGF-β
Physcion (μM) - - 12.5 25 - -
Glycyrrhizic acid (μM) - - - - 100 -
MCC950 (μM) - - - - - 10
HMGB1
29 kDa
NLRP3
110 kDa
GSDMD
32 kDa
F4/80
160 kDa
GAPDH
37 kDa
kDa
180
140
100
75
60
45
35
25
15
10
15% Tris-Glycine
GAPDH
HMGB1
NLRP3
GSDMD
F4/80
75

Fig 9
